# The Systemic Inflammation Response Index as an Independent Predictor of Survival in Breast Cancer Patients: A Retrospective Study

**DOI:** 10.3389/fmolb.2022.856064

**Published:** 2022-02-28

**Authors:** Mengliu Zhu, Li Chen, Xiangyi Kong, Xiangyu Wang, Yi Fang, Xingrui Li, Jing Wang

**Affiliations:** ^1^ Department of Breast Surgical Oncology, National Cancer Center/National Clinical Research Center for Cancer/Cancer Hospital, Chinese Academy of Medical Sciences and Peking Union Medical College, Beijing, China; ^2^ Department of Thyroid and Breast Surgery, Tongji Hospital, Tongji Medical College of Huazhong University of Science and Technology, Wuhan, China

**Keywords:** breast cancer, neoadjuvant chemotherapy, systemic inflammation response index (SIRI), prognosis, disease-free survival (DFS), overall survival (OS)

## Abstract

There is a close relationship between inflammatory cells and tumors, but the pathways that connect the two remain unclear. This research explores the clinical and prognostic value of the systemic inflammation response index (SIRI) in breast cancer patients. The study included 477 breast cancer patients who underwent neoadjuvant chemotherapy and 308 breast cancer patients who did not in our center between January 1998 and December 2016. Optimal SIRI threshold values were determined using the receiver operating characteristic curve (ROC). Patients were then reclassified as SIRI ≥0.80 group (High SIRI group) and SIRI <0.80 group (Low SIRI group). The outcomes were analyzed by statistical methods. The univariate and multivariate analyses demonstrated that SIRI independently predicted survival in breast cancer. The disease-free survival (DFS) and overall survival (OS) in patients with low SIRI scores were significantly longer in contrast to those with high SIRI scores (41.50 vs. 37.63 months, and 64.57 vs. 58.42 months). Further subgroup analyses revealed that low SIRI score patients who also had either early breast cancer, advanced breast cancer, or different molecular subtypes also possessed longer mean survival time of DFS and OS in contrast to those with high SIRI levels (*χ*2 = 2.379, *p* = 0.123, and *χ*2 = 5.153, *p* = 0.023; *χ*2 = 11.080, *p* = 0.0009 and *χ*2 = 15.900, *p* < 0.0001; *χ*2 = 16.020, *p* < 0.0001 and *χ*2 = 22.050, *p* < 0.0001, respectively). SIRI serves as an easily accessible, replicable, and minimally invasive prognostic tool in breast cancer patients. Lower SIRI scores were predictive of a longer DFS and OS after surgery in breast cancer patients. SIRI may serve as a marker to guide clinical management and prognostication of breast cancer.

## Introduction

Breast cancer is among the most frequently diagnosed cancers in females. This malignancy exerts a deleterious effect on patient quality of life and is a significant public health issue (Dan et al., 2020). The GLOBOCAN 2018 Research reports that there are more than 2 million new cases of breast cancer annually, with more than 600,000 deaths due to breast cancer occurring each year. There is a concerning trend towards a younger age of the first diagnosis, along with an overall higher number of breast cancer cases (Bray et al., 2020). Recent data in China shows a marked rise in breast cancer incidence, especially in its developed coastal cities. Experts predict that breast cancer incidences in China are expected to reach a staggering 100 cases per 100,000 postmenopausal women in the future (Li et al., 2019). Despite the comprehensiveness of current treatment modalities of breast cancer that includes surgery, adjuvant chemotherapy, radiotherapy, targeted therapy, immunotherapy, and Chinese medicine treatment, patient outcomes are still unsatisfactory (Chen et al., 2017).

The tumor microenvironment, which includes the extracellular matrix, stromal cells, lymphatic and blood vessels, as well as resident immune cells, has been found to be a key determinant in dictating tumor behavior. Of interest is the role of inflammation, which is postulated to be influential in tumor progression and metastasis (Singh et al., 2019). Recent studies have confirmed that various markers of the systemic inflammatory response, for example, the C-reactive Protein (CRP), Platelet to Lymphocyte Ratio (PLR), Lymphocyte to Monocyte Ratio (LMR), and Neutrophil to Lymphocyte Ratio (NLR), all correlate to the prognosis of a myriad of tumors such as high-grade glioma (He et al., 2021b), colorectal cancer (Dagmura et al., 2021), head and neck cancer (Saroul et al., 2021), oral squamous cell cancer (Yamagata et al., 2021), and gastric cancer (Liu et al., 2021). The latest evidence also suggests that a similar tumor-inflammation relationship exists for breast cancer, indicating that quantifying the inflammatory response may be useful in treating and prognosticating breast cancer (Dong et al., 2021). Common blood indices, including platelets (P), monocytes (M), neutrophils (N), hemoglobin (Hb), total red blood cell count (R), total white blood cell count (WBC), and serum albumin (ALB), along with its derivatives, NLR, MLR, LMR, PLR, D-NLR, prognostic nutritional value [PNI, 10 × serum ALB (g/dL) + 0.005 × total lymphocyte count], and SIRI (Neutrophil × Platelet/Lymphocyte) may all be reflective of malignant tumor states (Mantovani et al., 2008). Breast cancer is currently diagnosed by a combination of pathological assessments of tissue samples taken via core needle biopsy (CNB) and various imaging modalities including breast ultrasound, mammography, and magnetic resonance imaging (MRI) (Al-Hattali et al., 2019). Nevertheless, the concept of being able to prognosticate breast cancer based on routine peripheral blood examinations is attractive given the ease of access, replicability, and lower cost. This investigation seeks to determine the utility of common inflammatory markers in the context of breast cancer.

## Materials and Methods

### Study Population

Our study comprised 785 breast cancer patients. Of these, 477 underwent surgery and received neoadjuvant chemotherapy (NACT) in our center between January 1998 to December 2016 were included in our study. The control cohort comprised308 breast cancer patients who received surgical treatment only at the same center and during the same timeframe. All participants underwent routine examination and examination on admission, a comprehensive assessment of their condition, and provided written informed consent prior to study inclusion. All patients were diagnosed by CNB or histopathology. TNM staging was carried out in accordance with the eighth edition AJCC (American Joint Committee on Cancer) and the Union for International Cancer Control (UICC) (Weigelt and Reis-Filho, 2009; Cserni et al., 2018).

### Inclusion and Exclusion Criteria

The inclusion criterion was as follows: 1) Breast cancer was confirmed by CNB or pathological examination; 2) Zubrod-Ecog-WHO (ZPS) between 0 and 2 and Karnofsky Performance Scores (KPS) ≥80; 3) Expected to survive more than 3 months; 4) Patients did not receive anti-tumor treatment before admission, including chemotherapy, radiotherapy, immunotherapy, interventional therapy, and traditional Chinese medicine treatment; 5) Surgery was performed after the completion of NACT; 6) Admission examination showed no obvious abnormalities in liver, kidney, lung, heart, brain, and bone marrow; 7) Inpatient medical records and postoperative follow-up data were complete.

The following was our exclusion criteria: 1) The possibility of distant organ metastasis was not able to be excluded on imaging examinations such as abdominal B-ultrasound, chest Computed Tomography (CT), and breast MRI, or the breast tumor was not able to be resected due to the definite presence of metastasis; 2) Patients received anti-tumor therapy, such as radiotherapy, chemotherapy, and targeted therapy; 3) The presence of serious comorbidities that were refractory to treatment such as hypertension, heart disease, and diabetes; 4) Advanced breast cancer, including breast cancer ulcers, inflammatory breast cancer, and infected tumors; 5) Blood transfusion history within 1 month before receiving NACT; 6) Patients who were poorly compliant and not cooperative with treatment.

### Chemotherapy Regimen

The NACT treatment regimen included anthracyclines and/or taxanes. Protocols used included the AC regimen, ACF regimen, CT regimen, ACT regimen, AT regimen, and TP regimen.

### Peripheral Venous Blood Collection Method

All patients took an early morning fasting peripheral venous blood sample of 2–5 ml. Peripheral venous blood specimens were obtained within 7 days before surgery in patients without neoadjuvant chemotherapy. And others were obtained within 7 days before neoadjuvant chemotherapy. WBC, neutrophils, hemoglobin, lymphocytes, monocytes, platelets, eosinophils, basophils, and other hematological parameters in peripheral venous blood were evaluated using the XE-2100 hematology analyzer (Sysmex, KOBE, Japan). SIRI was calculated based on the following formula: (neutrophils×monocytes)/lymphocyte count .

### Evaluation Assays

The size of the tumor, invasion depth, and the degree of lymph node metastasis were determined by breast ultrasound, mammography, and MRI. Tumor diameters were taken as their largest measurable diameter. The eighth edition of AJCC guided TNM staging (Weigelt and Reis-Filho, 2009; Cserni et al., 2018). The main pathological types of breast cancer were invasive lobular carcinoma, invasive ductal carcinoma, and other types. Molecular classification of breast cancer were triple-negative breast cancers, HER2 overexpressing tumors, Luminal B/HER-2-negative, Luminal B/HER2-positive, and Luminal A types (He et al., 2021a). The Miller and Payne histological grade (MPG) allowed for evaluation of the reduction of tumor cells after NACT and is divided into five grades (Therasse et al., 2000). The efficacy of NACT on tumor lesions after treatment was done in accordance with the 2000 RECIST criteria (Amat et al., 2002). The histological classification of breast cancer is based on the Nottingham Joint Histological Classification (Elston and Ellis modification of the Scarff-Bloom-Richardson grading protocol) (Kaba et al., 2004). NACT toxicity and adverse effects were assessed based on the National Cancer Institute Common Toxicity Criteria (NCI-CTC) (Diakos et al., 2014).

### Follow-Up

Follow-up was performed according to the NCCN (2020) guidelines: 1) every 3 months for 1–2 years postoperatively, 2) every 6 months for 3–5 years postoperatively, and 3) every year after 5 years until death. Disease-Free Survival (DFS) was the duration between postoperative day 1 until tumor recurrence, distant metastasis, or death from other causes. The duration between postoperative day 1 until the last follow-up or death was defined as Overall Survival (OS). The duration between postoperative day 1 until death or the last follow-up was deemed as survival.

### Statistical Methods

SPSS 17.0 (version 17.0; SPSS Inc., Chicago, IL, United States) and GraphPad Prism Software (Version 8.0; GraphPad Inc., La Jolla, CA, United States) were used to carry out all statistical analyses. The critical optimal threshold values of related variables were identified utilizing receiver operating characteristic curves (ROC), while the area under the curve (AUC) value was used to evaluate the prognostic accuracy. Qualitative data was depicted in terms of the number of cases (%), with intergroup comparisons carried out *via* the *χ* ^2^ test or Fisher’s exact test. OS was determined *via* the Kaplan-Meier test. The survival rate between the two groups was contrasted with the log-rank method. Univariate and multivariate Cox proportional hazards regression models were used to discern potential prognostic factors. The association between various parameters and breast cancer prognosis was determined using hazard ratios (HRs) and 95% confidence intervals (CIs). A two-tailed *p* value of less than 0.05 was interpreted as achieving statistical significance.

## Results

### SIRI is Predictive of Clinical Outcomes in Breast Cancer Before Neoadjuvant Chemotherapy

We applied the ROC curve to confirm that the optimal SIRI threshold was 0.80. Based on the optimal threshold, two SIRI groups were formed: SIRI <0.80 group (Low SIRI group) and SIRI ≥0.80 group (High SIRI group). All enrolled patients were female between ages 22–82 years. The average age of 47 ± 10 years, and the median age of 47 years 756 patients (96.31%) were married, and 29 patients (3.69%) were unmarried. BMI ranged from 16.36 to 38.19, with a median BMI of 24.00 and a mean BMI of 24.45 ± 3.55. 292 patients were postmenopausal (37.20%), and 493 patients were premenopausal (62.80%). ABO blood group distribution showed that there were 214 patients with type A (27.26%), 262 patients with type B (33.38%), 234 patients with type O (29.81%), and 75 patients with type AB (9.55%). All patients received surgical treatment, among which 606 cases (77.20%) underwent total resection of breast cancer and 179 cases (22.80%) underwent breast-conserving surgery. There were 758 cases of ductal carcinoma (96.56%), 13 cases of lobular carcinoma (1.66%), and 14 cases of other types of breast cancer (1.78%). The histological classification of breast cancer included 133 cases of grade I (16.94%), 431 cases of grade II (54.90%), and 221 cases of grade III (28.15%). There were 516 cases (65.73%) who received postoperative chemotherapy and 269 cases (34.27%) who did not receive postoperative chemotherapy. 483 cases (61.53%) received endocrine therapy after breast cancer surgery, and 302 cases (38.47%) did not receive endocrine therapy. 202 cases (25.73%) received targeted therapy after breast cancer surgery, while 583 cases (74.27%) did not receive targeted therapy. The clinical data of 785 breast cancer patients are depicted in Table 1.

1) In all breast cancer patients, there were 484 cases in the low SIRI group and 301 cases in the high SIRI group. Statistical analysis showed that BMI (*χ*2 = 4.801, *p* = 0.028), clinical T stage (*χ*2 = 19.137, *p* = 0.0007), clinical N stage (*χ*2 = 14.841, *p* = 0.005), clinical TNM stage (*χ*2 = 12.114, *p* = 0.002), postoperative chemotherapy regimen (*χ*2 = 16.590, *p* = 0.005), postoperative chemotherapy (*χ*2 = 10.404, *p* = 0.001), postoperative chemotherapy times (*χ*2 = 13.066, *p* = 0.0003), and postoperative targeted therapy (*χ*2 = 9.697, *p* = 0.002) demonstrated statistically significant differences between the two SIRI groups.
2) In the NACT group (477 patients), there were 267 cases in the low SIRI group and 210 cases in the high SIRI group. Statistical analysis showed that clinical T stage (*χ*2 = 10.284, *p* = 0.036), neoadjuvant chemotherapy regimen (*χ*2 = 46.320, *p* < 0.0001), postoperative chemotherapy (*χ*2 = 9.882, *p* = 0.043), postoperative chemotherapy times (*χ*2 = 5.320, *p* = 0.021) and postoperative targeted (*χ*2 = 4.153, *p* = 0.042) were statistically significant.
3) In the non-NACT group (308 breast cancer patients), there were 217 cases in the low SIRI group and 91 cases in the high SIRI group. Statistical analysis showed that postoperative chemotherapy (*χ*2 = 13.250, *p* = 0.021) was statistically significant.

**TABLE 1:** Demographic and clinicopathologic characteristics of 785 patients with breast cancer.

Parameters	N	SIRI 785				N	SIRI 477				N	SIRI 308			
Cases (n)	785	Low SIRI 484	High SIRI 301	χ2	*p* value		Low SIRI 267	High SIRI 210	χ2	*p* value		Low SIRI 217	High SIRI 91	χ2	*p* value
Age (years)				0.193	0.660				0.054	0.816				1.504	0.220
<47	386 (49.17%)	235 (48.55%)	151 (50.17%)			230 (48.22%)	130 (48.69%)	100 (47.62%)			156 (50.65%)	105 (48.39%)	51 (56.04%)		
≥47	399 (50.83%)	249 (51.45%)	150 (49.83%)			247 (51.78%)	137 (51.31%)	110 (52.38%)			152 (49.35%)	112 (51.61%)	40 (43.96%)		
Marital status				0.117	0.732				0.690	0.406				3.013	0.083
Married	756 (96.31%)	467 (96.49%)	289 (96.01%)			457 (95.81%)	254 (95.13%)	203 (96.67%)			299 (97.08%)	213 (98.16%)	86 (94.51%)		
Unmarried	29 (3.69%)	17 (3.51%)	12 (3.99%)			20 (4.19%)	13 (4.87%)	7 (3.33%)			9 (2.92%)	4 (1.84%)	5 (5.49%)		
Occupation				3.276	0.194				0.133	0.936				7.681	0.022
Mental worker	358 (45.61%)	226 (46.69%)	132 (43.85%)			238 (49.90%)	135 (50.56%)	103 (49.05%)			120 (38.96%)	91 (41.94%)	29 (31.87%)		
Manual worker	125 (15.92%)	83 (17.15%)	42 (13.95%)			66 (13.84%)	37 (13.86%)	29 (13.81%)			59 (19.16%)	46 (21.20%)	13 (14.29%)		
Others	302 (38.47%)	175 (36.16%)	127 (42.19%)			173 (36.27%)	95 (35.58%)	78 (37.14%)			129 (41.88%)	80 (36.87%)	49 (53.85%)		
Weight (kg)				1.014	0.314				0.677	0.411				0.465	0.495
<62.00	383 (48.79%)	243 (50.21%)	140 (46.51%)			235 (49.27%)	136 (50.94%)	99 (47.14%)			148 (48.05%)	107 (49.31%)	41 (45.05%)		
≥62.00	402 (51.21%)	241 (49.79%)	161 (53.49%)			242 (50.73%)	131 (49.06%)	111 (52.86%)			160 (51.95%)	110 (50.69%)	50 (54.95%)		
Height (m)				1.696	0.193				0.036	0.850				2.244	0.134
<1.60	337 (42.93%)	199 (41.12%)	138 (45.85%)			218 (45.70%)	121 (45.32%)	97 (46.19%)			119 (38.64%)	78 (35.94%)	41 (45.05%)		
≥1.60	448 (57.07%)	285 (58.88%)	163 (54.15%)			259 (54.30%)	146 (54.68%)	113 (53.81%)			189 (61.36%)	139 (64.06%)	50 (54.95%)		
BMI				4.801	0.028				2.674	0.102				3.186	0.074
<24.00	391 (49.81%)	256 (52.89%)	135 (44.85%)			245 (51.36%)	146 (54.68%)	99 (47.14%)			146 (47.40%)	110 (50.69%)	36 (39.56%)		
≥24.00	394 (50.19%)	228 (47.11%)	166 (55.15%)			232 (48.64%)	121 (45.32%)	111 (52.86%)			162 (52.60%)	107 (49.31%)	55 (60.44%)		
Menarche age (year)				1.076	0.300				0.484	0.487				0.246	0.620
<14	308 (39.24%)	183 (37.81%)	125 (41.53%)			196 (41.09%)	106 (39.70%)	90 (42.86%)			112 (36.36%)	77 (35.48%)	35 (38.46%)		
≥14	477 (60.76%)	301 (62.19%)	176 (58.47%)			281 (58.91%)	161 (60.30%)	120 (57.14%)			196 (63.64%)	140 (64.52%)	56 (61.54%)		
Menopause				1.119	0.290				2.674	0.102				0.083	0.773
No	493 (62.80%)	297 (61.36%)	196 (65.12%)			280 (58.70%)	148 (55.43%)	132 (62.86%)			213 (69.16%)	149 (68.66%)	64 (70.33%)		
Yes	292 (37.20%)	187 (38.64%)	105 (34.88%)			197 (41.30%)	119 (44.57%)	78 (37.14%)			95 (30.84%)	68 (31.34%)	27 (29.67%)		
ABO blood type				2.449	0.654				4.406	0.354				2.856	0.582
A	214 (27.26%)	129 (26.65%)	85 (28.24%)			132 (27.67%)	68 (25.47%)	64 (30.48%)			82 (26.62%)	61 (28.11%)	21 (23.08%)		
B	262 (33.38%)	168 (34.71%)	94 (31.23%)			145 (30.40%)	83 (31.09%)	62 (29.52%)			117 (37.99%)	85 (39.17%)	32 (35.16%)		
O	234 (29.81%)	146 (30.17%)	88 (29.24%)			146 (30.61%)	90 (33.71%)	56 (26.67%)			88 (28.57%)	56 (25.81%)	32 (35.16%)		
AB	75 (9.55%)	41 (8.47%)	34 (11.30%)			54 (11.32%)	26 (9.74%)	28 (13.33%)			21 (6.82%)	15 (6.91%)	6 (6.59%)		
Tumor site				0.049	0.824				1.404	0.236				2.417	0.120
Right	369 (47.01%)	226 (46.69%)	143 (47.51%)			233 (48.85%)	124 (46.44%)	109 (51.90%)			136 (44.16%)	102 (47.00%)	34 (37.36%)		
Left	416 (52.99%)	258 (53.31%)	158 (52.49%)			244 (51.15%)	143 (53.56%)	101 (48.10%)			172 (55.84%)	115 (53.00%)	57 (62.64%)		
Clinical T stage				19.137	0.001				10.284	0.036				3.161	0.531
T1	168 (21.40%)	113 (23.35%)	68 (22.59%)			65 (13.63%)	43 (16.10%)	22 (10.48%)			103 (33.44%)	70 (32.26%)	33 (36.26%)		
T2	413 (52.61%)	269 (55.58%)	132 (43.85%)			226 (47.38%)	133 (49.81%)	93 (44.29%)			187 (60.71%)	136 (62.67%)	51 (56.04%)		
T3	131 (16.69%)	71 (14.67%)	59 (19.60%)			115 (24.11%)	62 (23.22%)	53 (25.24%)			16 (5.19%)	9 (4.15%)	7 (7.69%)		
T4	73 (9.30%)	31 (6.40%)	42 (13.95%)			71 (14.88%)	29 (10.86%)	42 (20.00%)			2 (0.65%)	2 (0.92%)	0 (0.00%)		
Clinical N stage				14.841	0.005				0.665	0.956				5.613	0.230
N0	299 (38.09%)	210 (43.39%)	90 (29.90%)			73 (15.30%)	44 (16.48%)	29 (13.81%)			226 (73.38%)	166 (76.50%)	60 (65.93%)		
N1	233 (29.68%)	135 (27.89%)	97 (32.23%)			164 (34.38%)	90 (33.71%)	74 (35.24%)			69 (22.40%)	45 (20.74%)	24 (26.37%)		
N2	160 (20.38%)	88 (18.18%)	72 (23.92%)			151 (31.66%)	84 (31.46%)	67 (31.90%)			9 (2.92%)	4 (1.84%)	5 (5.49%)		
N3	93 (11.85%)	51 (10.54%)	42 (13.95%)			89 (18.66%)	49 (18.35%)	40 (19.05%)			4 (1.30%)	2 (0.92%)	2 (2.20%)		
Clinical TNM stage				12.114	0.002				1.930	0.381				0.555	0.758
I	92 (11.72%)	66 (13.64%)	26 (8.64%)			14 (2.94%)	10 (3.75%)	4 (1.90%)			78 (25.32%)	56 (25.81%)	22 (24.18%)		
II	382 (48.66%)	248 (51.24%)	134 (44.52%)			168 (35.22%)	97 (36.33%)	71 (33.81%)			214 (69.48%)	151 (69.59%)	63 (69.23%)		
III	311 (39.62%)	170 (35.12%)	141 (46.84%)			295 (61.84%)	160 (59.93%)	135 (64.29%)			16 (5.19%)	10 (4.61%)	6 (6.59%)		
Neoadjuvant Chemotherapy															
Chemotherapy regimen									46.320	<0.0001					
EC/ECF						28 (5.87%)	21 (7.87%)	7 (3.33%)							
CT/ECT						27 (5.66%)	21 (7.87%)	6 (2.86%)							
ET						223 (46.75%)	131 (49.06%)	92 (43.81%)							
TP						141 (29.56%)	61 (22.85%)	80 (38.10%)							
Others						58 (12.16%)	33 (12.36%)	25 (11.90%)							
Chemotherapy times									3.407	0.065					
<6						134 (28.09%)	84 (31.46%)	50 (23.81%)							
≥6						343 (71.91%)	183 (68.54%)	160 (76.19%)							
Response									1.326	0.857					
CR						7 (1.47%)	6 (2.25%)	1 (0.48%)							
PR						312 (65.41%)	169 (63.30%)	143 (68.10%)							
SD						151 (31.66%)	86 (32.21%)	65 (30.95%)							
PD						7 (1.47%)	6 (2.25%)	1 (0.48%)							
Miller and Payne grade									9.371	0.053					
1						22 (4.61%)	11 (4.12%)	11 (5.24%)							
2						126 (26.42%)	70 (26.22%)	56 (26.67%)							
3						177 (37.11%)	112 (41.95%)	65 (30.95%)							
4						62 (13.00%)	26 (9.74%)	36 (17.14%)							
5						90 (18.87%)	48 (17.98%)	42 (20.00%)							
Pathological response									0.024	0.876					
pCR						72 (15.09%)	40 (14.98%)	32 (15.24%)							
non-pCR						405 (84.91%)	229 (85.77%)	176 (83.81%)							
Post-chemotherapy regimen				16.590	0.005				6.457	0.264				13.250	0.021
EC/ECF	125 (15.92%)	88 (18.18%)	37 (12.29%)			43 (9.01%)	25 (9.36%)	18 (8.57%)			82 (26.62%)	63 (29.03%)	19 (20.88%)		
CT/ECT	125 (15.92%)	75 (15.50%)	50 (16.61%)			30 (6.29%)	20 (7.49%)	10 (4.76%)			95 (30.84%)	55 (25.35%)	40 (43.96%)		
ET	97 (12.36%)	71 (14.67%)	26 (8.64%)			37 (7.76%)	25 (9.36%)	12 (5.71%)			60 (19.48%)	46 (21.20%)	14 (15.38%)		
TP	61 (7.77%)	37 (7.64%)	24 (7.97%)			39 (8.18%)	23 (8.61%)	16 (7.62%)			22 (7.14%)	14 (6.45%)	8 (8.79%)		
Others	108 (13.76%)	68 (14.05%)	40 (13.29%)			81 (16.98%)	48 (17.98%)	33 (15.71%)			27 (8.77%)	20 (9.22%)	7 (7.69%)		
NO	269 (34.27%)	145 (29.96%)	124 (41.20%)			247(51.78%)	126 (47.19%)	121 (57.62%)			22 (7.14%)	19 (8.76%)	3 (3.30%)		
Type of surgery				0.082	0.775				0.037	0.848				0.654	0.419
Mastectomy	606 (77.20%)	372 (76.86%)	234 (77.74%)			406 (85.12%)	228 (85.39%)	178 (84.76%)			200 (64.94%)	144 (66.36%)	56 (61.54%)		
Breast-conserving surgery	179 (22.80%)	112 (23.14%)	67 (22.26%)			71 (14.88%)	39 (14.61%)	32 (15.24%)			108 (35.06%)	73 (33.64%)	35 (38.46%)		
Tumor size (cm)				0.785	0.675				0.512	0.774				0.016	0.992
≤2 cm	437 (55.67%)	267 (55.17%)	170 (56.48%)			263 (55.14%)	144 (53.93%)	119 (56.67%)			174 (56.49%)	123 (56.68%)	51 (56.04%)		
>2 and <5 cm	299 (38.09%)	189 (39.05%)	110 (36.54%)			172 (36.06%)	100 (37.45%)	72 (34.29%)			127 (41.23%)	89 (41.01%)	38 (41.76%)		
≥5 cm	49 (6.24%)	28 (5.79%)	21 (6.98%)			42 (8.81%)	23 (8.61%)	19 (9.05%)			7 (2.27%)	5 (2.30%)	2 (2.20%)		
Histologic type				1.481	0.477				0.906	0.636				3.556	0.169
Ductal	758 (96.56%)	470 (97.11%)	288 (95.68%)			461 (96.65%)	258 (96.63%)	203 (96.67%)			297 (96.43%)	212 (97.70%)	85 (93.41%)		
Lobular	13 (1.66%)	6 (1.24%)	7 (2.33%)			7 (1.47%)	3 (1.12%)	4 (1.90%)			6 (1.95%)	3 (1.38%)	3 (3.30%)		
Others	14 (1.78%)	8 (1.65%)	6 (1.99%)			9 (1.89%)	6 (2.25%)	3 (1.43%)			5 (1.62%)	2 (0.92%)	3 (3.30%)		
Histologic grade				3.881	0.144				3.327	0.190				5.327	0.070
I	133 (16.94%)	76 (15.70%)	57 (18.94%)			108 (22.64%)	54 (20.22%)	54 (25.71%)			25 (8.12%)	22 (10.14%)	3 (3.30%)		
II	431 (54.90%)	279 (57.64%)	152 (50.50%)			244 (51.15%)	146 (54.68%)	98 (46.67%)			187 (60.71%)	133 (61.29%)	54 (59.34%)		
III	221 (28.15%)	129 (26.65%)	92 (30.56%)			125 (26.21%)	67 (25.09%)	58 (27.62%)			96 (31.17%)	62 (28.57%)	34 (37.36%)		
Pathological TNM classification															
Pathological T stage				4.021	0.403				2.050	0.727				1.824	0.768
Tis/T0	92 (11.72%)	50 (10.33%)	42 (13.95%)			88 (18.45%)	46 (17.23%)	42 (20.00%)			4 (1.30%)	4 (1.84%)	0 (0.00%)		
T1	302 (38.47%)	187 (38.64%)	115 (38.21%)			190 (39.83%)	108 (40.45%)	82 (39.05%)			112 (36.36%)	79 (36.41%)	33 (36.26%)		
T2	326 (41.53%)	208 (42.98%)	118 (39.20%)			149 (31.24%)	85 (31.84%)	64 (30.48%)			177 (57.47%)	123 (56.68%)	54 (59.34%)		
T3	45 (5.73%)	29 (5.99%)	16 (5.32%)			34 (7.13%)	21 (7.87%)	13 (6.19%)			11 (3.57%)	8 (3.69%)	3 (3.30%)		
T4	20 (2.55%)	10 (2.07%)	10 (3.32%)			16 (3.35%)	7 (2.62%)	9 (4.29%)			4 (1.30%)	3 (1.38%)	1 (1.10%)		
Pathological N stage				2.054	0.726				1.523	0.823				1.628	0.804
N0	326 (41.53%)	201 (41.53%)	125 (41.53%)			176 (36.90%)	96 (35.96%)	80 (38.10%)			150 (48.70%)	105 (48.39%)	45 (49.45%)		
N1	175 (22.29%)	115 (23.76%)	60 (19.93%)			101 (21.17%)	62 (23.22%)	39 (18.57%)			74 (24.03%)	53 (24.42%)	21 (23.08%)		
N2	122 (15.54%)	71 (14.67%)	51 (16.94%)			77 (16.14%)	42 (15.73%)	35 (16.67%)			45 (14.61%)	29 (13.36%)	16 (17.58%)		
N3	162 (20.64%)	97 (20.04%)	65 (21.59%)			123 (25.79%)	67 (25.09%)	56 (26.67%)			39 (12.66%)	30 (13.82%)	9 (9.89%)		
Pathological TNM stage				2.384	0.666				1.795	0.773				1.621	0.805
Tis/T0	74 (9.43%)	43 (8.88%)	31 (10.30%)			71 (14.88%)	40 (14.98%)	31 (14.76%)			3 (0.97%)	3 (1.38%)	0 (0.00%)		
I	157 (20.00%)	96 (19.83%)	61 (20.27%)			83 (17.40%)	44 (16.48%)	39 (18.57%)			74 (24.03%)	52 (23.96%)	22 (24.18%)		
II	262 (33.38%)	171 (35.33%)	91 (30.23%)			118 (24.74%)	72 (26.97%)	46 (21.90%)			144 (46.75%)	99 (45.62%)	45 (49.45%)		
III	292 (37.20%)	174 (35.95%)	118 (39.20%)			205 (42.98%)	111 (41.57%)	94 (44.76%)			87 (28.25%)	63 (29.03%)	24 (26.37%)		
Total lymph nodes				0.204	0.652				2.866	0.091				0.047	0.829
<21	391 (49.81%)	238 (49.17%)	153 (50.83%)			202 (42.35%)	104 (38.95%)	98 (46.67%)			189 (61.36%)	134 (61.75%)	55 (60.44%)		
≥21	394 (50.19%)	246 (50.83%)	148 (49.17%)			275 (57.65%)	163 (61.05%)	112 (53.33%)			119 (38.64%)	83 (38.25%)	36 (39.56%)		
Positive lymph nodes				0.103	0.749				0.175	0.676				0.109	0.742
<1	329 (41.91%)	205 (42.36%)	124 (41.20%)			179 (37.53%)	98 (36.70%)	81 (38.57%)			150 (48.70%)	107 (49.31%)	43 (47.25%)		
≥1	456 (58.09%)	279 (57.64%)	177 (58.80%)			298 (62.47%)	169 (63.30%)	129 (61.43%)			158 (51.30%)	110 (50.69%)	48 (52.75%)		
Postoperative complications				0.002	0.968				0.017	0.898				0.375	0.540
No	728 (92.74%)	449 (92.77%)	279 (92.69%)			449 (94.13%)	251 (94.01%)	198 (94.29%)			279 (90.58%)	198 (91.24%)	81 (89.01%)		
Yes	57 (7.26%)	35 (7.23%)	22 (7.31%)			28 (5.87%)	16 (5.99%)	12 (5.71%)			29 (9.42%)	19 (8.76%)	10 (10.99%)		
Postoperative chemotherapy				10.404	0.001				5.120	0.024				2.881	0.090
No	269 (34.27%)	145 (29.96%)	124 (41.20%)			247 (51.78%)	126 (47.19%)	121 (57.62%)			22 (7.14%)	19 (8.76%)	3 (3.30%)		
Yes	516 (65.73%)	339 (70.04%)	177 (58.80%)			230 (48.22%)	141 (52.81%)	89 (42.38%)			286 (92.86%)	198 (91.24%)	88 (96.70%)		
Postoperative chemotherapy times				13.066	0.0003				5.320	0.021				1.473	0.225
<4	374 (47.64%)	206 (42.56%)	168 (55.81%)			340 (71.28%)	179 (67.04%)	161 (76.67%)			34 (11.04%)	27 (12.44%)	7 (7.69%)		
≥4	411 (52.36%)	278 (57.44%)	133 (44.19%)			137 (28.72%)	88 (32.96%)	49 (23.33%)			274 (88.96%)	190 (87.56%)	84 (92.31%)		
Postoperative radiotherapy				0.496	0.481				0.118	0.732				2.750	0.097
No	196 (24.97%)	125 (25.83%)	71 (23.59%)			119 (24.95%)	65 (24.34%)	54 (25.71%)			77 (25.00%)	60 (27.65%)	17 (18.68%)		
Yes	589 (75.03%)	359 (74.17%)	230 (76.41%)			358 (75.05%)	202 (75.66%)	156 (74.29%)			231 (75.00%)	157 (72.35%)	74 (81.32%)		
Postoperative endocrine therapy				1.927	0.165				0.059	0.808				1.563	0.211
No	302 (38.47%)	177 (36.57%)	125 (41.53%)			206 (43.19%)	114 (42.70%)	92 (43.81%)			96 (31.17%)	63 (29.03%)	33 (36.26%)		
Yes	483 (61.53%)	307 (63.43%)	176 (58.47%)			271 (56.81%)	153 (57.30%)	118 (56.19%)			212 (68.83%)	154 (70.97%)	58 (63.74%)		
Postoperative targeted therapy				9.697	0.002				4.153	0.042				2.753	0.097
No	583 (74.27%)	378 (78.10%)	205 (68.11%)			332 (69.60%)	196 (73.41%)	136 (64.76%)			251 (81.49%)	182 (83.87%)	69 (75.82%)		
Yes	202 (25.73%)	106 (21.90%)	96 (31.89%)			145 (30.40%)	71 (26.59%)	74 (35.24%)			57 (18.51%)	35 (16.13%)	22 (24.18%)		

### Hematological Parameters

Breast cancer patient nutritional statuses were evaluated using several parameters, with their median values shown in brackets: ALB (45.2 g/L), blood glucose (GLU) (5.33 mmol/L), alkaline phosphatase (ALP) (64.00 U/L), *γ*-glutamyl transpeptidase (GGT) (17.00 U/L), lactate dehydrogenase (LDH) (167.00 U/L), alanine aminotransferase (ALT) (15.00 U/L), and aspartate aminotransferase (AST) (18.00 U/L).

The following are other parameters obtained with their respective median values shown in brackets: CRP (0.20 mg/dl), carbohydrate antigen 125 (CA125) (13.35 U/mL), carbohydrate antigen (CA15-3) (11.63 U/mL), carcinoembryonic antigen (CEA) (1.66 ng/ml), plasma D-dimer (D-D) (0.29 mg/L), fibrinogen (FIB) (2.85 g/L), international standardized ratio of prothrombin time (INR) (0.93), fibrinogen degradation products (FDP) (1.40 µg/mL), and W (6.01 × 10^9^/L), R (4.40 × 10^12^/L), Hb (132 g/L), N (3.68 × 10^9^/L), L (1.76 × 10^9^/L), M (0.35 × 10^9^/L), E (0.06 × 10^9^/L), B (0.02 × 109/L), and P (243 × 109/L).

1) In all breast cancer patients, the parameters of LDH (*χ* ^2^ = 4.337, *p* = 0.037), CRP (*χ* ^2^ = 17.198, *p* < 0.0001), CA125 (*χ* ^2^ = 5.051, *p* = 0.025), FIB (*χ* ^2^ = 14.320, *p* < 0.0001), *p* = 0.0002, INR (*χ* ^2^ = 4.218, *p* = 0.040), FDP (*χ* ^2^ = 4.691, *p* = 0.030), W (*χ* ^2^ = 75.436, *p* < 0.0001), R (*χ* ^2^ = 7.107, *p* = 0.008), Hb (*χ* ^2^ = 7.361, *p* = 0.007), N (*χ* ^2^ = 142.491, *p* < 0.0001), L (*χ* ^2^ = 7.843, *p* = 0.005), M (*χ* ^2^ = 124.109, *p* < 0.0001), B (*χ* ^2^ = 9.429, *p* = 0.002), P (*χ* ^2^ = 13.231, *p* < 0.0001), L (*χ* ^2^ = 7.843, *p* < 0.0001), *p* = 0.0003 were statistically significant between high and low SIRI groups. The results are shown in Table 2.
2) In the NACT group (477 patients), FIB (*χ* ^2^ = 11.241, *p* = 0.0008), W (*χ* ^2^ = 57.819, *p* < 0.0001), R (*χ* ^2^ = 5.283, *p* = 0.022), Hb (*χ* ^2^ = 4.887, *p* = 0.027), N (*χ* ^2^ = 98.716, *p* < 0.0001), M (*χ* ^2^ = 100.469, *p* < 0.0001) and P (*χ* ^2^ = 8.329, *p* = 0.004) were statistically significant.
3) In the non-NACT group (308 breast cancer patients), ALB (*χ* ^2^ = 9.576, *p* = 0.002), CRP (*χ* ^2^ = 11.798, *p* = 0.0006), D-D (*χ* ^2^ = 5.007, *p* = 0.025), W (*χ* ^2^ = 20.949, *p* < 0.0001), Hb (*χ* ^2^ = 4.100, *p* = 0.043), N (*χ* ^2^ = 42.839, *p* < 0.0001), L (*χ* ^2^ = 4.817, *p* = 0.028), M (*χ* ^2^ = 26.521, *p* < 0.0001), E (*χ* ^2^ = 6.697, *p* = 0.010) and B (*χ* ^2^ = 9.248, *p* = 0.002) were statistically significant.

**TABLE 2:** The correlations between nutritional parameters/blood parameters and SIRI.

Parameters	N	SIRI 785				N	SIRI 477				N	SIRI 308			
Cases (*n*)	785	Low SIRI 484	High SIRI 301	χ2	*p* value		Low SIRI 267	High SIRI 210	χ2	*p* value		Low SIRI 217	High SIRI 91	χ2	*p* value
ALT (U/L)				0.820	0.365				0.071	0.791				1.699	0.192
<15	370 (47.13%)	234 (48.35%)	136 (45.18%)			208 (43.61%)	115 (43.07%)	93 (44.29%)			162 (52.60%)	119 (54.84%)	43 (47.25%)		
≥15	416 (52.99%)	250 (51.65%)	166 (55.15%)			269 (56.39%)	152 (56.93%)	117 (55.71%)			147 (47.73%)	98 (45.16%)	49 (53.85%)		
AST (U/L)				0.092	0.762				0.153	0.696				0.444	0.505
<18	378 (48.15%)	231 (47.73%)	147 (48.84%)			211 (44.23%)	116 (43.45%)	95 (45.24%)			167 (54.22%)	115 (53.00%)	52 (57.14%)		
≥18	407 (51.85%)	253 (52.27%)	154 (51.16%)			266 (55.77%)	151 (56.55%)	115 (54.76%)			141 (45.78%)	102 (47.00%)	39 (42.86%)		
LDH (U/L)				4.337	0.037				3.509	0.061				0.056	0.813
<167	376 (47.90%)	246 (50.83%)	130 (43.19%)			193 (40.46%)	118 (44.19%)	75 (35.71%)			183 (59.42%)	128 (58.99%)	55(60.44%)		
≥167	409 (52.10%)	238 (49.17%)	171 (56.81%)			284 (59.54%)	149 (55.81%)	135 (64.29%)			125 (40.58%)	89 (41.01%)	36 (39.56%)		
GGT (U/L)				2.314	0.128				1.413	0.235				0.084	0.772
<17	366 (46.62%)	236 (48.76%)	130 (43.19%)			203 (42.56%)	120 (44.94%)	83 (39.52%)			163 (52.92%)	116 (53.46%)	47 (51.65%)		
≥17	419 (53.38%)	248 (51.24%)	171 (56.81%)			274 (57.44%)	147 (55.06%)	127 (60.48%)			145 (47.08%)	101 (46.54%)	44 (48.35%)		
ALP (U/L)				0.273	0.601				2.149	0.143				1.369	0.242
<64	377 (48.03%)	236 (48.76%)	141 (46.84%)			227 (47.59%)	135 (50.56%)	92 (43.81%)			150 (48.70%)	101 (46.54%)	49 (53.85%)		
≥64	408 (51.97%)	248 (51.24%)	160 (53.16%)			250 (52.41%)	132 (49.44%)	118 (56.19%)			158 (51.30%)	116 (53.46%)	42 (46.15%)		
GLU (mmol/L)				0.093	0.761				0.002	0.962				0.013	0.909
<5.33	391 (49.81%)	239 (49.38%)	152 (50.50%)			247 (51.78%)	138 (51.69%)	109 (51.90%)			144 (46.75%)	101 (46.54%)	43 (47.25%)		
≥5.33	394 (50.19%)	245 (50.62%)	149 (49.50%)			230 (48.22%)	129 (48.31%)	101 (48.10%)			164 (53.25%)	116 (53.46%)	48 (52.75%)		
ALB (g/L)				3.817	0.051				0.007	0.933				9.576	0.002
<45.2	392 (49.94%)	255 (52.69%)	137 (45.51%)			235 (49.27%)	132 (49.44%)	103 (49.05%)			157 (50.97%)	123 (56.68%)	34 (37.36%)		
≥45.2	393 (50.06%)	229 (47.31%)	164 (54.49%)			242 (50.73%)	135 (50.56%)	107 (50.95%)			151 (49.03%)	94 (43.32%)	57 (62.64%)		
CRP (mg/dl)				17.198	<0.0001				2.475	0.116				11.798	0.001
<0.02	384 (48.92%)	265 (54.75%)	119 (39.53%)			187 (39.20%)	113 (42.32%)	74 (35.24%)			197 (63.96%)	152 (70.05%)	45 (49.45%)		
≥0.02	401 (51.08%)	219 (45.25%)	182 (60.47%)			290 (60.80%)	154 (57.68%)	136 (64.76%)			111 (36.04%)	65 (29.95%)	46 (50.55%)		
CA125 (U/ml)				5.051	0.025				2.956	0.086				0.784	0.376
<13.35	392 (49.94%)	257 (53.10%)	135 (44.85%)			221 (46.33%)	133 (49.81%)	88 (41.90%)			171 (55.52%)	124 (57.14%)	47 (51.65%)		
≥13.35	393 (50.06%)	227 (46.90%)	166 (55.15%)			256 (53.67%)	134 (50.19%)	122 (58.10%)			137 (44.48%)	93 (42.86%)	44 (48.35%)		
CA153 (U/ml)				0.236	0.627				0.723	0.395				2.060	0.151
<11.63	392 (49.94%)	245 (50.62%)	147 (48.84%)			208 (43.61%)	121 (45.32%)	87 (41.43%)			184 (59.74%)	124 (57.14%)	60 (65.93%)		
≥11.63	393 (50.06%)	239 (49.38%)	154 (51.16%)			269 (56.39%)	146 (54.68%)	123 (58.57%)			124 (40.26%)	93 (42.86%)	31 (34.07%)		
CEA (ng/ml)				2.025	0.155				2.025	0.155				2.174	0.140
<1.66	392 (49.94%)	232 (47.93%)	160 (53.16%)			212 (44.44%)	111 (41.57%)	101 (48.10%)			180 (58.44%)	121 (55.76%)	59 (64.84%)		
≥1.66	393 (50.06%)	252 (52.07%)	141 (46.84%)			265 (55.56%)	156 (58.43%)	109 (51.90%)			128 (41.56%)	96 (44.24%)	32 (35.16%)		
D-D (mg/L)				0.147	0.702				0.039	0.844				5.007	0.025
<0.29	387 (49.30%)	236 (48.76%)	151 (50.17%)			200 (41.93%)	113 (42.32%)	87 (41.43%)			187 (60.71%)	123 (56.68%)	64 (70.33%)		
≥0.29	398 (50.70%)	248 (51.24%)	150 (49.83%)			277 (58.07%)	154 (57.68%)	123 (58.57%)			121 (39.29%)	94 (43.32%)	27 (29.67%)		
FIB (g/L)				14.320	0.0002				11.241	0.001				1.468	0.226
<2.85	388 (49.43%)	265 (54.75%)	123 (40.86%)			216 (45.28%)	139 (52.06%)	77 (36.67%)			172 (55.84%)	126 (58.06%)	46 (50.55%)		
≥2.85	397 (50.57%)	219 (45.25%)	178 (59.14%)			261 (54.72%)	128 (47.94%)	133 (63.33%)			136 (44.16%)	91 (41.94%)	45 (49.45%)		
INR				4.218	0.040				0.884	0.347				0.425	0.515
<0.93	365 (46.50%)	239 (49.38%)	126 (41.86%)			177 (37.11%)	104 (38.95%)	73 (34.76%)			188 (61.04%)	135 (62.21%)	53 (58.24%)		
≥0.93	420 (53.50%)	245 (50.62%)	175 (58.14%)			300 (62.89%)	163 (61.05%)	137 (65.24%)			120 (38.96%)	82 (37.79%)	38 (41.76%)		
FDP (ug/ml)				4.691	0.030				0.300	0.584				2.025	0.155
<1.40	367 (46.75%)	241 (49.79%)	126 (41.86%)			137 (28.72%)	74 (27.72%)	63 (30.00%)			230 (74.68%)	167 (76.96%)	63 (69.23%)		
≥1.40	418 (53.25%)	243 (50.21%)	175 (58.14%)			340 (71.28%)	193 (72.28%)	147 (70.00%)			78 (25.32%)	50 (23.04%)	28 (30.77%)		
White blood cell (W) (×10^9^/L)				75.436	<0.0001				57.819	<0.0001				20.949	<0.0001
<6.01	389 (49.55%)	299 (61.78%)	90 (29.90%)			239 (50.10%)	175 (65.54%)	64 (30.48%)			150 (48.70%)	124 (57.14%)	26 (28.57%)		
≥6.01	396 (50.45%)	185 (38.22%)	211 (70.10%)			238 (49.90%)	92 (34.46%)	146 (69.52%)			158 (51.30%)	93 (42.86%)	65 (71.43%)		
Red blood cell (R) (×10^12^/L)				7.107	0.008				5.283	0.022				1.887	0.170
<4.40	389 (49.55%)	258 (53.31%)	131 (43.52%)			235 (49.27%)	144 (53.93%)	91 (43.33%)			154 (50.00%)	114 (52.53%)	40 (43.96%)		
≥4.40	396 (50.45%)	226 (46.69%)	170 (56.48%)			242 (50.73%)	123 (46.07%)	119 (56.67%)			154 (50.00%)	103 (47.47%)	51 (56.04%)		
Hemoglobin (Hb) (×10^9^/L)				7.361	0.007				4.887	0.027				4.100	0.043
<132	382 (48.66%)	254 (52.48%)	128 (42.52%)			243 (50.94%)	148 (55.43%)	95 (45.24%)			139 (45.13%)	106 (48.85%)	33 (36.26%)		
≥132	403 (51.34%)	230 (47.52%)	173 (57.48%)			234 (49.06%)	119 (44.57%)	115 (54.76%)			169 (54.87%)	111 (51.15%)	58 (63.74%)		
Neutrophil (N) (×10^9^/L)				142.491	<0.0001				98.716	<0.0001				42.839	<0.0001
<3.68	392 (49.94%)	323 (66.74%)	69 (22.92%)			229 (48.01%)	182 (68.16%)	47 (22.38%)			163 (52.92%)	141 (64.98%)	22 (24.18%)		
≥3.68	393 (50.06%)	161 (33.26%)	232 (77.08%)			248 (51.99%)	85 (31.84%)	163 (77.62%)			145 (47.08%)	76 (35.02%)	69 (75.82%)		
Lymphocyte (L) (×10^9^/L)				7.843	0.005				1.884	0.170				4.817	0.028
<1.76	391 (49.81%)	222 (45.87%)	169 (56.15%)			258 (54.09%)	137 (51.31%)	121 (57.62%)			133 (43.18%)	85 (39.17%)	48 (52.75%)		
≥1.76	394 (50.19%)	262 (54.13%)	132 (43.85%)			219 (45.91%)	130 (48.69%)	89 (42.38%)			175 (56.82%)	132 (60.83%)	43 (47.25%)		
Monocyte (M) (×10^9^/L)				124.109	<0.0001				100.469	<0.0001				26.521	<0.0001
<0.35	367 (46.75%)	302 (62.40%)	65 (21.59%)			216 (45.28%)	175 (65.54%)	41 (19.52%)			151 (49.03%)	127 (58.53%)	24 (26.37%)		
≥0.35	418 (53.25%)	182 (37.60%)	236 (78.41%)			261 (54.72%)	92 (34.46%)	169 (80.48%)			157 (50.97%)	90 (41.47%)	67 (73.63%)		
Eosinophils (E) (×10^9^/L)				3.395	0.065				0.041	0.839				6.697	0.010
<0.06	356 (45.35%)	207 (42.77%)	149 (49.50%)			241 (50.52%)	136 (50.94%)	105 (50.00%)			115 (37.34%)	71 (32.72%)	44 (48.35%)		
≥0.06	429 (54.65%)	277 (57.23%)	152 (50.50%)			236 (49.48%)	131 (49.06%)	105 (50.00%)			193 (62.66%)	146 (67.28%)	47 (51.65%)		
Basophils (B) (×10^9^/L)				9.429	0.002				2.588	0.108				9.248	0.002
<0.02	224 (28.54%)	157 (32.44%)	67 (22.26%)			136 (28.51%)	84 (31.46%)	52 (24.76%)			88 (28.57%)	73 (33.64%)	15 (16.48%)		
≥0.02	561 (71.46%)	327 (67.56%)	234 (77.74%)			341 (71.49%)	183 (68.54%)	158 (75.24%)			220 (71.43%)	144 (66.36%)	76 (83.52%)		
Platelet (P) (×10^9^/L)				13.231	0.0003				8.329	0.004				3.482	0.062
<243	388 (49.43%)	264 (54.55%)	124 (41.20%)			224 (46.96%)	141 (52.81%)	83 (39.52%)			164 (53.25%)	123 (56.68%)	41 (45.05%)		
≥243	397 (50.57%)	220 (45.45%)	177 (58.80%)			253 (53.04%)	126 (47.19%)	127 (60.48%)			144 (46.75%)	94 (43.32%)	50 (54.95%)		

### Survival Analysis Based on Univariate and Multivariate Cox Regression Survival Analyses

Through univariate analysis, we found that menopausal status, GLU, CA125, M, E, SIRI, histological type, pathological N stage, molecular type, Ki-67, CK5/6, lymph vessel invasion (LVI), postoperative targeted therapy, postoperative endocrine therapy, and postoperative chemotherapy were independent factors for improving DFS and OS. After multivariate analysis, we found that menopausal status, blood glucose, CA125, CA153, M, E, SIRI, histological grade, clinical N stage, pathological N and TNM stages, Ki-67, CK5/6, E-cadherin (E-cad), LVI, postoperative chemotherapy, and postoperative targeted therapy were independent factors for improving DFS and OS. Table 3 depicts all of the above results.

**TABLE 3:** Survival analyses based on univariate and multivariate Cox regression methods for predicting breast cancer patient DFS and OS.

		DFS				OS		
	Univariate analysis		Multivariate analysis		Univariate analysis		Multivariate analysis	
Parameters	Hazard ratio (95%CI)	*p* value	Hazard ratio (95%CI)	*p* value	Hazard ratio (95%CI)	*p* value	Hazard ratio (95%CI)	*p* value
Menopause		0.011		0.001		0.007		0.014
No	1 (reference)		1 (reference)		1 (reference)		1 (reference)	
Yes	1.598 (1.113–2.295)		1.487 (1.180–1.873)		1.392 (1.094–1.771)		1.344 (1.063–1.700)	
GLU (mmol/L)		0.003		0.006		0.013		0.018
<5.33	1 (reference)		1 (reference)		1 (reference)		1 (reference)	
≥5.33	0.662 (0.502–0.872)		0.732 (0.585–0.915)		0.692 (0.518–0.924)		0.749 (0.590–0.952)	
CA125 (U/ml)		0.013		0.026		0.018		0.049
<13.35	1(reference)		1 (reference)		1 (reference)		1 (reference)	
≥13.35	1.395 (1.073–1.813)		1.295 (1.032–1.624)		1.330 (1.050–1.685)		1.261 (1.001–1.589)	
CA153 (U/ml)		0.073				0.002		0.012
<11.63	1 (reference)				1 (reference)		1 (reference)	
≥11.63	1.291 (0.976–1.708)				1.554 (1.171–2.063)		1.331 (1.065–1.664)	
Neutrophil (N)×10^9^/L		0.482				0.278		
<3.68	1 (reference)				1 (reference)			
≥3.68	0.875 (0.603–1.269)				0.806 (0.545–1.190)			
Lymphocyte (L)×10^9^/L		0.481				0.412		
<1.76	1 (reference)				1 (reference)			
≥1.76	0.898 (0.668–1.209)				1.133 (0.840–1.527)			
Monocyte (M)×10^9^/L		0.004		<0.0001		<0.0001		<0.0001
<0.35	1 (reference)		1 (reference)		1 (reference)		1 (reference)	
≥0.35	1.419 (1.118–1.799)		1.627 (1.275–2.078)		1.869 (1.396–2.503)		1.637 (1.269–2.110)	
Eosinophils (E)×10^9^/L		0.015		0.008		0.001		0.010
<0.06	1 (reference)		1 (reference)		1 (reference)		1 (reference)	
≥0.06	0.717 (0.548–0.937)		0.740 (0.592–0.925)		0.636 (0.483–0.839)		0.744 (0.594–0.932)	
Platelet (P)×10^9^/L		0.137				0.304		
<243	1 (reference)				1 (reference)			
≥243	0.839 (0.666–1.058)				0.874 (0.678–1.128)			
Systemic inflammation response index (SIRI)		0.016		0.013		<0.0001		<0.0001
<112	1 (reference)		1 (reference)		1 (reference)		1 (reference)	
≥112	1.461 (1.074–1.988)		1.475 (1.085–2.005)		1.970 (1.431–2.712)		1.637 (1.269–2.110)	
Clinical stage								
Clinical N stage		0.230				0.001		<0.0001
N0	1 (reference)				1 (reference)		1 (reference)	
N1	0.934 (0.622–1.401)				1.532 (1.101–2.132)		1.371 (1.053–1.786)	
N2	0.883 (0.439–1.777)				1.704 (1.010–2.934)		1.400 (1.010–1.942)	
N3	1.476 (0.689–3.160)				3.525 (1.852–6.708)		3.034 (2.080–4.427)	
Histologic type		0.021		0.028		0.002		0.017
Ductal	1 (reference)		1 (reference)		1 (reference)		1 (reference)	
Lobular	2.581 (1.129–5.899)		2.495 (1.096–5.683)		3.006 (1.255–7.198)		1.943 (1.064–4.019)	
Others	2.046 (1.083–4.537)		1.987 (1.115–4.405)		2.948 (1.332–6.522)		2.357 (1.140–4.870)	
Pathological TNM classification								
Pathological N stage		0.014		<0.0001		0.0002		<0.0001
N0	1 (reference)		1 (reference)		1 (reference)		1 (reference)	
N1	2.901 (1.031–8.668)		1.518 (1.148–2.008)		2.001 (1.493–5.981)		1.330 (1.004–1.776)	
N2	3.928 (1.004–15.47)		1.499 (1.077–2.086)		6.029 (1.702–21.35)		1.495 (1.061–2.105)	
N3	6.219 (1.574–24.56)		1.897 (1.420–2.535)		10.24 (2.861–36.69)		2.006 (1.465–2.748)	
Pathological TNM stage		0.255				0.006		0.012
Tis/T0	1 (reference)				1 (reference)		1 (reference)	
I	2.662 (0.732–9.671)				2.600 (1.399–9.454)		1.986 (1.126–3.503)	
II	3.251 (0.862–12.26)				3.626 (1.043–13.70)		2.236 (1.098–4.844)	
III	1.998 (0.418–9.555)				2.532 (1.337–4.796)		2.645 (1.428–4.899)	
Positive lymph nodes		0.306				0.725		
<1	1 (reference)				1 (reference)			
≥1	0.509 (0.140–1.853)				0.788 (0.210–2.959)			
Postoperative pathology (IHC)								
Molecular subtype		0.018		0.029		0.097		
Luminal A	1 (reference)		1 (reference)		1 (reference)			
Luminal B HER2+	0.395 (0.216–0.724)		0.391 (0.213–0.716)		0.259 (0.093–0.722)			
Luminal B HER2-	0.535 (0.330–0.868)		0.468 (0.287–0.763)		0.535 (0.307–0.933)			
HER2 enriched	0.357 (0.193–0.662)		0.429 (0.233–0.790)		0.287 (0.096–0.853)			
Triple negative	0.534 (0.309–0.924)		0.455 (0.262–0.790)		0.557 (0.271–1.145)			
ER status		0.105				0.725		
Negative	1 (reference)				1 (reference)			
Positive	0.658 (0.397–1.090)				0.913 (0.551–1.512)			
PR status		0.257				0.155		
Negative	1 (reference)				1 (reference)			
Positive	1.253 (0.847–1.854)				1.306 (0.903–1.887)			
HER2 status		0.101				0.182		
Negative (0--++)	1 (reference)				1 (reference)			
Positive (+++)	2.115 (0.864–5.178)				1.826 (0.754–4.420)			
Ki-67 status		0.003		0.005		0.004		0.010
Negative (≤14%)	1 (reference)		1 (reference)		1 (reference)		1 (reference)	
Positive (>14%)	1.687 (1.190–2.391)		1.650 (1.167–2.333)		1.662 (1.172–2.356)		1.576 (1.116–2.225)	
CK5/6 status		0.011		0.001		0.017		<0.0001
Negative	1 (reference)		1 (reference)		1 (reference)		1 (reference)	
Positive	1.786 (1.142–2.792)		1.752 (1.265–2.426)		1.769 (1.107–2.825)		1.919 (1.386–2.659)	
E-cad status		0.279				<0.0001		<0.0001
Negative	1 (reference)				1 (reference)		1 (reference)	
Positive	1.212 (0.855–1.719)				2.379 (1.622–3.490)		2.320 (1.709–3.150)	
Lymph vessel invasion		0.040		<0.0001		0.012		0.004
Negative	1 (reference)		1 (reference)		1 (reference)		1 (reference)	
Positive	1.406 (1.016–1.945)		1.636 (1.285–2.083)		1.523 (1.097–2.114)		1.458 (1.131–1.880)	
Postoperative chemotherapy		<0.0001		<0.0001		<0.0001		0.004
No	1 (reference)		1 (reference)		1 (reference)		1 (reference)	
Yes	2.182 (1.489–3.198)		1.636 (1.285–2.083)		2.000 (1.359–2.942)		1.458 (1.131–1.880)	
Postoperative radiotherapy		0.183				0.089		
No	1 (reference)				1 (reference)			
Yes	1.254 (0.898–1.751)				1.348 (0.955–1.901)			
Postoperative endocrine therapy		0.015		0.032		0.080		
No	1 (reference)		1 (reference)		1 (reference)			
Yes	1.544 (1.088–2.190)		1.388 (1.029–1.874)		1.301 (0.969–1.747)			
Postoperative targeted therapy		<0.0001		<0.0001		0.004		<0.0001
No	1 (reference)		1 (reference)		1 (reference)		1 (reference)	
Yes	2.608 (1.799–3.781)		2.105 (1.638–2.706)		1.709 (1.188–2.456)		1.791 (1.397–2.296)	

### Disease-Free Survival and Overall Survival

**FIGURE 1:**
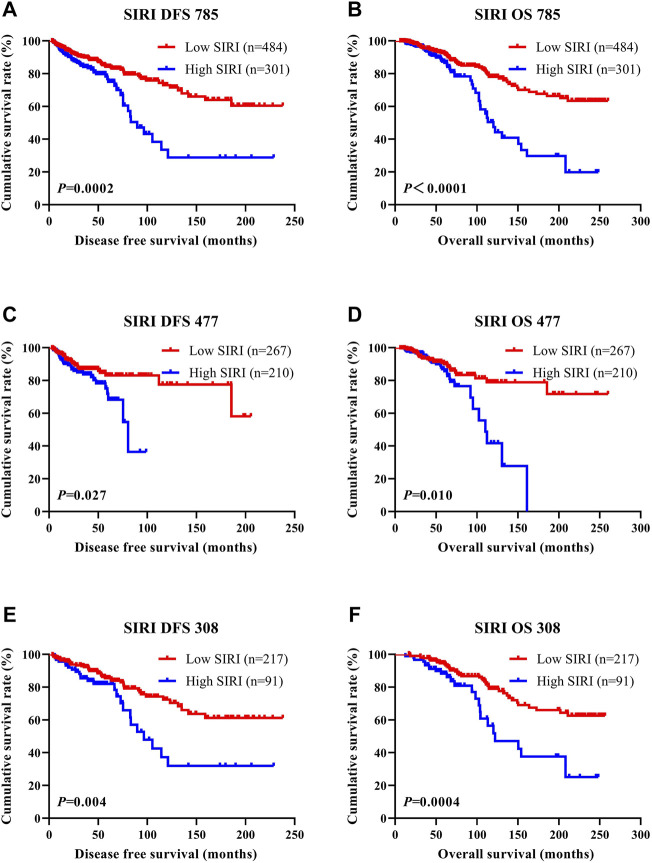
DFS and OS of breast cancer patients. DFS and OS of breast cancer patients. **(A)** Kaplan-Meier analysis of DFS for the SIRI of all patients with breast cancer. **(B)** Kaplan-Meier analysis of OS for the SIRI of all patients with breast cancer. **(C)** Kaplan-Meier analysis of DFS for the SIRI of patients with breast cancer (NACT group). **(D)** Kaplan-Meier analysis of OS for the SIRI of patients with breast cancer (NACT group). **(E)** Kaplan-Meier analysis of DFS for the SIRI of patients with breast cancer (non-NACT group). **(F)** Kaplan-Meier analysis of OS for the SIRI of patients with breast cancer (non-NACT group).

SIRI was found to be an independent factor that improved DFS and OS on both univariate and multivariate analyses, and the optimal threshold value for SIRI was 0.80. Univariate analysis demonstrated that low SIRI significantly improved DFS and OS (HR: 1.461, 95% CI: 1.074–1.988, *p* = 0.016 and HR: 1.475, 95% CI: 1.085–2.005, *p* = 0.013). Multivariate analysis showed that a low SIRI significantly improved DFS and OS (HR: 1.970, 95% CI: 1.431–2.712, *p* < 0.0001 and HR: 1.637, 95% CI: 1.269–2.110, *p* < 0.0001). Patients with low SIRI scores had mean survival times of DFS and OS of 41.50 months (3.10–238.00 months) and 64.57 months (6.43–260.00 months), respectively. The average DFS and OS survival time of SIRI in the high group was 37.63 months (3.13–238.00 months) and 58.42 months (10.77–256.40 months), respectively. The log-rank analysis shown that the average DFS and OS survival time of SIRI in the low group were remarkably longer in contrast to that of SIRI in the high group (*χ* ^2^ = 14.290, *p* = 0.0002, and *χ* ^2^ = 20.690, *p* < 0.0001), as shown in Figure 1.

### The Association Between SIRI Scores and Tumor Node Metastasis (TNM) Stage

The N stage was an independent predictor of DFS and OS, as revealed by univariate and multivariate analyses. The pathological TNM stage is an independent factor of OS. The ability of SIRI to determine breast cancer prognosis was further assessed by examining the relationship between SIRI and the TNM stage. Early breast cancer was determined to be pathological stages Tis/T0 and I, while advanced breast cancer was pathological stages II and III. Both early and advanced forms of breast cancer were subjected to log-rank analysis to determine their respective DFS and OS.

**FIGURE 2:**
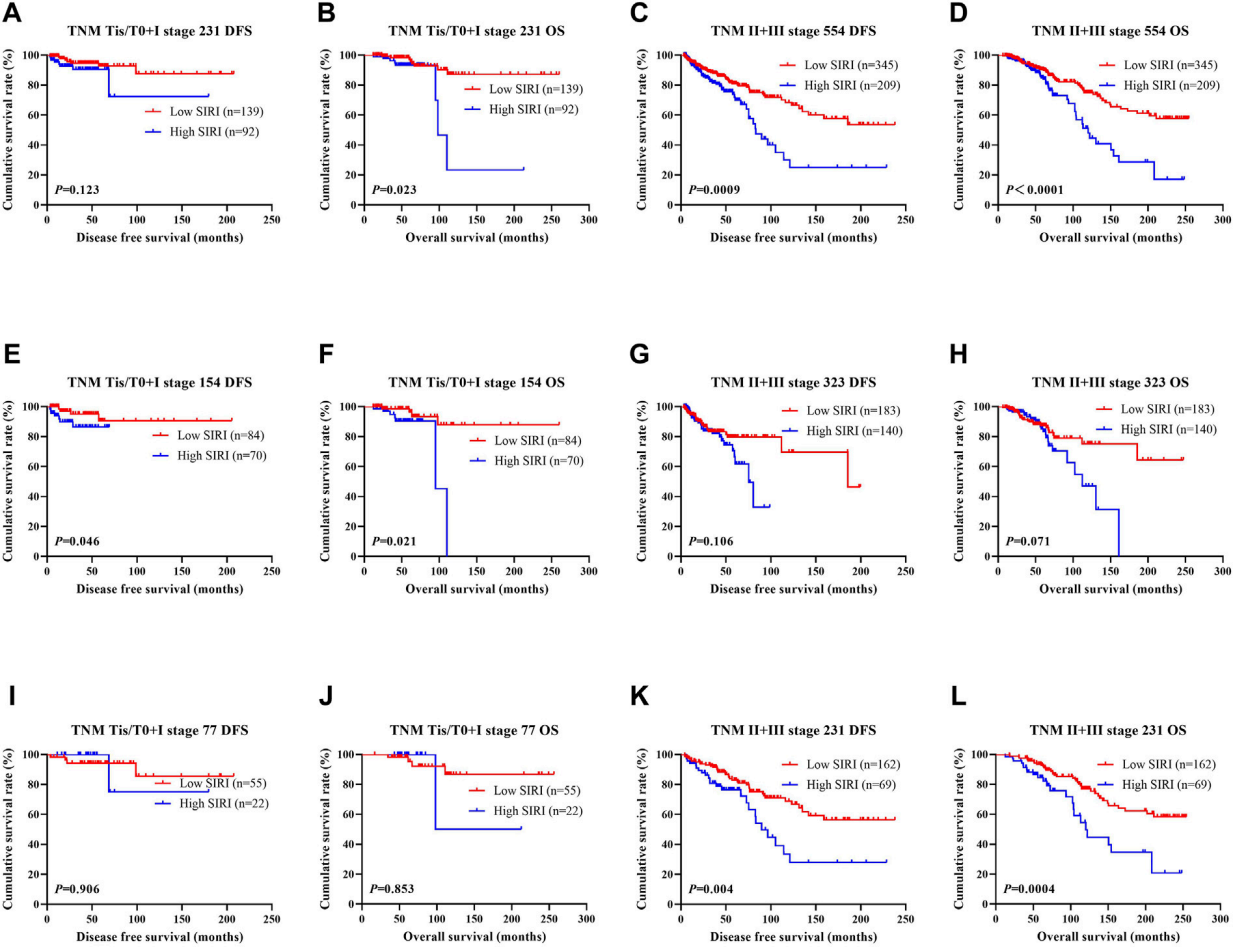
DFS and OS based on SIRI scores of patients with breast cancer of different pathological stage. DFS and OS based on SIRI scores of patients with breast cancer of different pathological stage. **(A)** Kaplan-Meier analysis of DFS for the SIRI of patients with early breast cancer. **(B)** Kaplan-Meier analysis of OS for the SIRI of patients with early breast cancer. **(C)** Kaplan-Meier analysis of DFS for the SIRI of patients with advanced breast cancer. **(D)** Kaplan-Meier analysis of OS for the SIRI of patients with advanced breast cancer. **(E)** Kaplan-Meier analysis of DFS for the SIRI of patients with early breast cancer (NACT group). **(F)** Kaplan-Meier analysis of OS for the SIRI of patients with early breast cancer (NACT group). **(G)** Kaplan-Meier analysis of DFS for the SIRI of patients with advanced breast cancer (NACT group). **(H)** Kaplan-Meier analysis of OS for the SIRI of patients with advanced breast cancer (NACT group). **(I)** Kaplan-Meier analysis of DFS for the SIRI of patients with early breast cancer (non-NACT group). **(J)** Kaplan-Meier analysis of OS for the SIRI of patients with early breast cancer (non-NACT group). **(K)** Kaplan-Meier analysis of DFS for the SIRI of patients with advanced breast cancer (non-NACT group). **(L)** Kaplan-Meier analysis of OS for the SIRI of patients with advanced breast cancer (non-NACT group).

Early breast cancer patients and low SIRI scores had notably longer DFS and OS in contrast to those high SIRI score patients (*χ* ^2^ = 2.379, *p* = 0.123, and *χ* ^2^ = 5.153, *p* = 0.023), as shown in Figure 2A and Figure 2B. 2). Similarly, patients with advanced breast cancer and low SIRI scores also had remarkably longer average DFS and OS in contrast to patients with elevated SIRI scores (*χ* ^2^ = 11.080, *p* = 0.0009 and *χ* ^2^ = 15.900, *p* < 0.0001), as shown in Figure 2C and Figure 2D. The DFS and OS of SIRI and TNM stage of the NACT and non-NACT cohorts are shown in Figures 2E–L, respectively.

### The Association Between Systemic Inflammatory Response Index Scores and Breast Cancer Molecular Subtype

We found that the molecular subtype of breast cancer was an independent risk factor of DFS based on univariate and multivariate analyses. Of the 785 patients with breast cancer, 171 cases were triple-negative type, 98 cases were Luminal B HER2-positive type, 325 cases were Luminal B HER2-negative type, 62 cases were Luminal A type, and 129 cases were HER2-overexpressing type. Table 4 shows the detailed information of the molecular type of breast cancer.

1) In all breast cancer patients, HER2 (*χ* ^2^ = 8.077, *p* = 0.005), E-cad (*χ* ^2^ = 21.406, *p* < 0.0001), epidermal growth factor receptor (EGFR) (*χ* ^2^ = 6.339, *p* = 0.012), topoisomerase (DNA) II alpha (TOP2A) (*χ* ^2^ = 5.595, *p* = 0.018), and LVI (*χ* ^2^ = 4.403, *p* = 0.036). were statistically significant.
2) In the NACT group (477 patients), there were no significant statistically between them.
3) In the non-NACT group (308 breast cancer patients), HER2 (*χ* ^2^ = 5.660, *p* = 0.017), E-cad (*χ* ^2^ = 14.686, *p* = 0.0001), EGFR (*χ* ^2^ = 6.983, *p* = 0.008), TOP2A (*χ* ^2^ = 8.526, *p* = 0.004) and LVI (*χ* ^2^ = 11.377, *p* = 0.007) were statistically significant.

**TABLE 4:** The relationship between SIRI scores and molecular breast cancer subtype.

Parameters	N	SIRI 785				N	SIRI 477				N	SIRI 308			
Cases (n)	785	Low SIRI 484	High SIRI 301	χ2	*p* value		Low SIRI 267	High SIRI 210	χ2	*p* value		Low SIRI 217	High SIRI 91	χ2	*p* value
Core needle biopsy (N = 477)															
Molecular subtype									3.520	0.475					
Luminal A						25 (5.24%)	15 (5.62%)	10 (4.76%)							
Luminal B HER2+						67 (14.05%)	31 (11.61%)	36 (17.14%)							
Luminal B HER2-						186 (38.99%)	105 (39.33%)	81 (38.57%)							
HER2 enriched						91 (19.08%)	51 (19.10%)	40 (19.05%)							
Triple negative						108 (22.64%)	65 (24.34%)	43 (20.48%)							
ER status									0.042	0.838					
Negative						191 (40.04%)	108 (40.45%)	83 (39.52%)							
Positive						286 (59.96%)	159 (59.55%)	127 (60.48%)							
ER status									0.929	0.920					
0–25%						228 (47.80%)	129 (48.31%)	99 (47.14%)							
26–50%						42 (8.81%)	26 (9.74%)	16 (7.62%)							
51–75%						33 (6.92%)	18 (6.74%)	15 (7.14%)							
76–100%						174 (36.48%)	94 (35.21%)	80 (38.10%)							
PR status									0.964	0.326					
Negative						189 (39.62%)	111 (41.57%)	78 (37.14%)							
Positive						288 (60.38%)	156 (58.43%)	132 (62.86%)							
PR status									2.467	0.651					
0–25%						286 (59.96%)	165 (61.80%)	121 (57.62%)							
26–50%						67 (14.05%)	35 (13.11%)	32 (15.24%)							
51–75%						45 (9.43%)	21 (7.87%)	24 (11.43%)							
76–100%						79 (16.56%)	46 (17.23%)	33 (15.71%)							
HER2 status									1.743	0.187					
Negative (0--++)						313 (65.62%)	182 (68.16%)	131 (62.38%)							
Positive (+++)						164 (34.38%)	85 (31.84%)	79 (37.62%)							
Ki-67 status									1.455	0.118					
Negative (≤14%)						84 (17.61%)	52 (19.48%)	32 (15.24%)							
Positive (>14%)						393 (82.39%)	215 (80.52%)	178 (84.76%)							
Ki-67 status									1.218	0.875					
0–25%						161 (33.75%)	92 (34.46%)	69 (32.86%)							
26–50%						189 (39.62%)	109 (40.82%)	80 (38.10%)							
51–75%						88 (18.45%)	45 (16.85%)	43 (20.48%)							
76–100%						39 (8.18%)	21 (7.87%)	18 (8.57%)							
Postoperative pathology (IHC)															
Molecular subtype				8.634	0.125				5.449	0.364				12.370	0.030
Luminal A	62 (7.90%)	41 (8.47%)	21 (6.98%)			41 (8.60%)	22 (8.24%)	19 (9.05%)			21 (6.82%)	19 (8.76%)	2 (2.20%)		
Luminal B HER2+	98 (12.48%)	52 (10.74%)	46 (15.28%)			61 (12.79%)	28 (10.49%)	33 (15.71%)			37 (12.01%)	24 (11.06%)	13 (14.29%)		
Luminal B HER2-	325 (41.40%)	211 (43.60%)	114 (37.87%)			166 (34.80%)	96 (35.96%)	70 (33.33%)			159 (51.62%)	115 (53.00%)	44 (48.35%)		
HER2 enriched	129 (16.43%)	70 (14.46%)	59 (19.60%)			96 (20.13%)	53 (19.85%)	43 (20.48%)			33 (10.71%)	17 (7.83%)	16 (17.58%)		
Triple negative	171 (21.78%)	110 (22.73%)	61 (20.27%)			113 (23.69%)	68 (25.47%)	45 (21.43%)			58 (18.83%)	42 (19.35%)	16 (17.58%)		
ER status				0.465	0.495				0.286	0.593				1.884	0.170
Negative	296 (37.71%)	178 (36.78%)	118 (39.20%)			195 (40.88%)	112 (41.95%)	83 (39.52%)			101 (32.79%)	66 (30.41%)	35 (38.46%)		
Positive	489 (62.29%)	306 (63.22%)	183 (60.80%)			282 (59.12%)	155 (58.05%)	127 (60.48%)			207 (67.21%)	151 (69.59%)	56 (61.54%)		
ER status				3.061	0.548				0.530	0.971				6.402	0.171
0–25%	375 (47.77%)	232 (47.93%)	143 (47.51%)			235 (49.27%)	134 (50.19%)	101 (48.10%)			140 (45.45%)	98 (45.16%)	42 (46.15%)		
26–50%	66 (8.41%)	41 (8.47%)	25 (8.31%)			31 (6.50%)	16 (5.99%)	15 (7.14%)			35 (11.36%)	25 (11.52%)	10 (10.99%)		
51–75%	48 (6.11%)	24 (4.96%)	24 (7.97%)			27 (5.66%)	14 (5.24%)	13 (6.19%)			21 (6.82%)	10 (4.61%)	11 (12.09%)		
76–100%	296 (37.71%)	187 (38.64%)	109 (36.21%)			184 (38.57%)	103 (38.58%)	81 (38.57%)			112 (36.36%)	84 (38.71%)	28 (30.77%)		
PR status				1.168	0.280				0.007	0.933				1.720	0.190
Negative	315 (40.13%)	187 (38.64%)	128 (42.52%)			210 (44.03%)	118 (44.19%)	92 (43.81%)			105 (34.09%)	69 (31.80%)	36 (39.56%)		
Positive	470 (59.87%)	297 (61.36%)	173 (57.48%)			267 (55.97%)	149 (55.81%)	118 (56.19%)			203 (65.91%)	148 (68.20%)	55 (60.44%)		
PR status				6.924	0.140				1.764	0.779				2.296	0.682
0–25%	502 (63.95%)	301 (62.19%)	201 (66.78%)			335 (70.23%)	187 (70.04%)	148 (70.48%)			167 (54.22%)	114 (52.53%)	53 (58.24%)		
26–50%	90 (11.46%)	57 (11.78%)	33 (10.96%)			48 (10.06%)	28 (10.49%)	20 (9.52%)			42 (13.64%)	29 (13.36%)	13 (14.29%)		
51–75%	55 (7.01%)	29 (5.99%)	26 (8.64%)			38 (7.97%)	18(6.74%)	20 (9.52%)			17 (5.52%)	11 (5.07%)	6 (6.59%)		
76–100%	138 (17.58%)	97 (20.04%)	41 (13.62%)			56 (11.74%)	34 (12.73%)	22 (10.48%)			82 (26.62%)	63 (29.03%)	19 (20.88%)		
HER2 status				8.077	0.005				1.824	0.177				5.660	0.017
Negative (0--++)	557 (70.96%)	361 (74.59%)	196 (65.12%)			320 (67.09%)	186 (69.66%)	134 (63.81%)			237 (76.95%)	175 (80.65%)	62 (68.13%)		
Positive (+++)	228 (29.04%)	123 (25.41%)	105 (34.88%)			157 (32.91%)	81 (30.34%)	76 (36.19%)			71 (23.05%)	42 (19.35%)	29 (31.87%)		
Ki-67 status				0.423	0.516				0.072	0.788				2.802	0.094
Negative (≤14%)	219 (27.90%)	139 (28.72%)	80 (26.58%)			153 (32.08%)	87 (32.58%)	66 (31.43%)			66 (21.43%)	52 (23.96%)	14 (15.38%)		
Positive (>14%)	566 (72.10%)	345 (71.28%)	221 (73.42%)			324 (67.92%)	180 (67.42%)	144 (68.57%)			242 (78.57%)	165 (76.04%)	77 (84.62%)		
Ki-67 status				5.107	0.277				4.227	0.376				1.436	0.838
0–25%	342 (43.57%)	215 (44.42%)	127 (42.19%)			233 (48.85%)	134 (50.19%)	99 (47.14%)			109 (35.39%)	81 (37.33%)	28 (30.77%)		
26–50%	257 (32.74%)	163 (33.68%)	94 (31.23%)			139 (29.14%)	81 (30.34%)	58 (27.62%)			118 (38.31%)	82 (37.79%)	36 (39.56%)		
51–75%	137 (17.45%)	83 (17.15%)	54 (17.94%)			70 (14.68%)	38 (14.23%)	32 (15.24%)			67 (21.75%)	45 (20.74%)	22 (24.18%)		
76–100%	49 (6.24%)	23 (4.75%)	26 (8.64%)			35 (7.34%)	14 (5.24%)	21 (10.00%)			14 (4.55%)	9 (4.15%)	5 (5.49%)		
AR status				1.209	0.272				0.018	0.892				0.040	0.841
Negative	666 (84.84%)	416 (85.95%)	250 (83.06%)			362 (75.89%)	202 (75.66%)	160 (76.19%)			304 (98.70%)	214 (98.62%)	90 (98.90%)		
Positive	119 (15.16%)	68 (14.05%)	51 (16.94%)			115 (24.11%)	65 (24.34%)	50 (23.81%)			4 (1.30%)	3 (1.38%)	1 (1.10%)		
AR status				1.665	0.797				3.144	0.534				0.021	0.885
0–25%	688 (87.64%)	424 (87.60%)	264 (87.71%)			383 (80.29%)	209 (78.28%)	174 (82.86%)			305 (99.03%)	215 (99.08%)	90 (98.90%)		
26–50%	25 (3.18%)	13 (2.69%)	12 (3.99%)			25 (5.24%)	13 (4.87%)	12 (5.71%)			0 (0.00%)	0 (0.00%)	0 (0.00%)		
51–75%	29 (3.69%)	20 (4.13%)	9 (2.99%)			29 (6.08%)	20 (7.49%)	9 (4.29%)			0 (0.00%)	0 (0.00%)	0 (0.00%)		
76–100%	43 (5.48%)	27 (5.58%)	16 (5.32%)			40 (8.39%)	25 (9.36%)	15 (7.14%)			3 (0.97%)	2 (0.92%)	1 (1.10%)		
CK5/6 status				1.336	0.248				0.940	0.332				0.003	0.954
Negative	684 (87.13%)	427 (88.22%)	257 (85.38%)			406 (85.12%)	231 (86.52%)	175 (83.33%)			278 (90.26%)	196 (90.32%)	82 (90.11%)		
Positive	101 (12.87%)	57 (11.78%)	44 (14.62%)			71 (14.88%)	36 (13.48%)	35 (16.67%)			30 (9.74%)	21 (9.68%)	9 (9.89%)		
E-cad status				21.406	<0.0001				3.593	0.058				14.686	0.0001
Negative	353 (44.97%)	249 (51.45%)	104 (34.55%)			170 (35.64%)	105 (39.33%)	65 (30.95%)			183 (59.42%)	144 (66.36%)	39 (42.86%)		
Positive	432 (55.03%)	235 (48.55%)	197 (65.45%)			307 (64.36%)	162 (60.67%)	145 (69.05%)			125 (40.58%)	73 (33.64%)	52 (57.14%)		
EGFR status				6.339	0.012				0.494	0.482				6.983	0.008
Negative	589 (75.03%)	378 (78.10%)	211 (70.10%)			335 (70.23%)	191 (71.54%)	144 (68.57%)			254 (82.47%)	187 (86.18%)	67 (73.63%)		
Positive	196 (24.97%)	106 (21.90%)	90 (29.90%)			142 (29.77%)	76 (28.46%)	66 (31.43%)			54 (17.53%)	30 (13.82%)	24 (26.37%)		
P53 status				0.642	0.423				0.303	0.582				0.528	0.467
Negative	395 (50.32%)	249 (51.45%)	146 (48.50%)			243 (50.94%)	139 (52.06%)	104 (49.52%)			152 (49.35%)	110 (50.69%)	42 (46.15%)		
Positive	390 (49.68%)	235 (48.55%)	155 (51.50%)			234 (49.06%)	128 (47.94%)	106 (50.48%)			156 (50.65%)	107 (49.31%)	49 (53.85%)		
P53 status				1.755	0.781				3.412	0.491				0.082	0.960
0–25%	576 (73.38%)	362 (74.79%)	214 (71.10%)			353 (74.00%)	204 (76.40%)	149 (70.95%)			223 (72.40%)	158 (72.81%)	65 (71.43%)		
26–50%	80 (10.19%)	49 (10.12%)	31 (10.30%)			45 (9.43%)	25 (9.36%)	20 (9.52%)			35 (11.36%)	24 (11.06%)	11 (12.09%)		
51–75%	108 (13.76%)	61 (12.60%)	47 (15.61%)			58 (12.16%)	26 (9.74%)	32 (15.24%)			50 (16.23%)	35 (16.13%)	15 (16.48%)		
76–100%	21 (2.68%)	12 (2.48%)	9 (2.99%)			21 (4.40%)	12 (4.49%)	9 (4.29%)			0 (0.00%)	0 (0.00%)	0 (0.00%)		
TOP2A status				5.595	0.018				0.101	0.750				8.526	0.004
Negative	299 (38.09%)	200 (41.32%)	99 (32.89%)			165 (34.59%)	94 (35.21%)	71 (33.81%)			134 (43.51%)	106 (48.85%)	28 (30.77%)		
Positive	486 (61.91%)	284 (58.68%)	202 (67.11%)			312 (65.41%)	173 (64.79%)	139 (66.19%)			174 (56.49%)	111 (51.15%)	63 (69.23%)		
TOP2A status				4.005	0.405				1.690	0.793				15.817	0.003
0–25%	575 (73.25%)	366 (75.62%)	209 (69.44%)			354 (74.21%)	200 (74.91%)	154 (73.33%)			221 (71.75%)	166 (76.50%)	55 (60.44%)		
26–50%	158 (20.13%)	90 (18.60%)	68 (22.59%)			88 (18.45%)	45 (16.85%)	43 (20.48%)			70 (22.73%)	45 (20.74%)	25 (27.47%)		
51–75%	49 (6.24%)	26 (5.37%)	23 (7.64%)			33 (6.92%)	21 (7.87%)	12 (5.71%)			16 (5.19%)	5 (2.30%)	11 (12.09%)		
76–100%	3 (0.38%)	2 (0.41%)	1 (0.33%)			2 (0.42%)	1 (0.37%)	1 (0.48%)			1 (0.32%)	1 (0.46%)	0 (0.00%)		
Lymph vessel invasion				4.403	0.036				0.048	0.826				11.377	0.001
Negative	558 (71.08%)	357 (73.76%)	201 (66.78%)			320 (67.09%)	178 (66.67%)	142 (67.62%)			238 (77.27%)	179 (82.49%)	59 (64.84%)		
Positive	227 (28.92%)	127 (26.24%)	100 (33.22%)			157 (32.91%)	89 (33.33%)	68 (32.38%)			70 (22.73%)	38 (17.51%)	32 (35.16%)		
Neural invasion				0.0004	0.984				0.470	0.493				0.059	0.808
Negative	670 (85.35%)	413 (85.33%)	257 (85.38%)			384 (80.50%)	212 (79.40%)	172 (81.90%)			286 (92.86%)	201 (92.63%)	85 (93.41%)		
Positive	115 (14.65%)	71 (14.67%)	44 (14.62%)			93 (19.50%)	55 (20.60%)	38 (18.10%)			22 (7.14%)	16 (7.37%)	6 (6.59%)		

**FIGURE 3:**
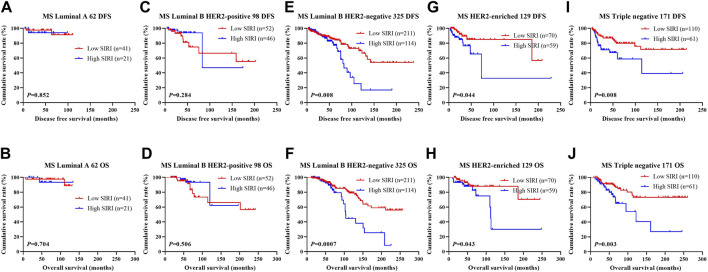
DFS and OS based on SIRI scores in patients with breast cancer of various molecular subtypes. DFS and OS based on SIRI scores in patients with breast cancer of various molecular subtypes. **(A)** Kaplan-Meier analysis of DFS for the SIRI of patients with luminal A breast cancer. **(B)** Kaplan-Meier analysis of OS for the SIRI of patients with luminal A breast cancer. **(C)** Kaplan-Meier analysis of DFS for the SIRI of patients with luminal B HER2-positive breast cancer. **(D)** Kaplan-Meier analysis of OS for the SIRI of patients with luminal B HER2-positive breast cancer. **(E)** Kaplan-Meier analysis of DFS for the SIRI of patients with luminal B HER2-negative breast cancer. **(F)** Kaplan-Meier analysis of OS for the SIRI of patients with luminal B HER2-negative breast cancer. **(G)** Kaplan-Meier analysis of DFS for the SIRI of patients with HER2-enriched breast cancer. **(H)** Kaplan-Meier analysis of OS for the SIRI of patients with HER2-enriched breast cancer. **(I)** Kaplan-Meier analysis of DFS for the SIRI of patients with triple-negative breast cancer. **(J)** Kaplan-Meier analysis of OS for the SIRI of patients with triple-negative breast cancer.

**FIGURE 4:**
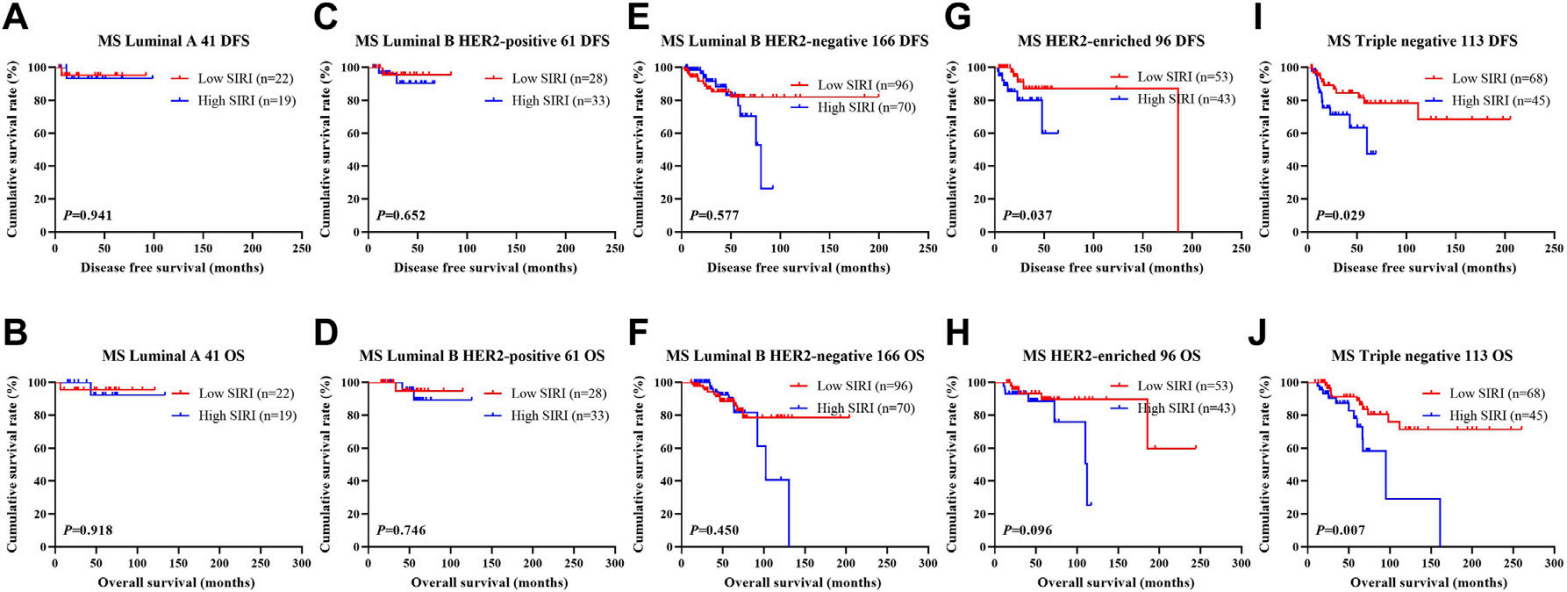
DFS and OS based on SIRI scores in patients with breast cancer of various molecular subtypes (NACT group). DFS and OS based on SIRI scores in patients with breast cancer of various molecular subtypes (NACT group). **(A)** Kaplan-Meier analysis of DFS for the SIRI of patients with luminal A breast cancer. **(B)** Kaplan-Meier analysis of OS for the SIRI of patients with luminal A breast cancer. **(C)** Kaplan-Meier analysis of DFS for the SIRI of patients with luminal B HER2-positive breast cancer. **(D)** Kaplan-Meier analysis of OS for the SIRI of patients with luminal B HER2-positive breast cancer. **(E)** Kaplan-Meier analysis of DFS for the SIRI of patients with luminal B HER2-negative breast cancer. **(F)** Kaplan-Meier analysis of OS for the SIRI of patients with luminal B HER2-negative breast cancer. **(G)** Kaplan-Meier analysis of DFS for the SIRI of patients with HER2-overexpressing breast cancer. **(H)** Kaplan-Meier analysis of OS for the SIRI of patients with HER2-overexpressing breast cancer. **(I)** Kaplan-Meier analysis of DFS for the SIRI of patients with triple-negative breast cancer. **(J)** Kaplan-Meier analysis of OS for the SIRI of patients with triple-negative breast cancer.

**FIGURE 5:**
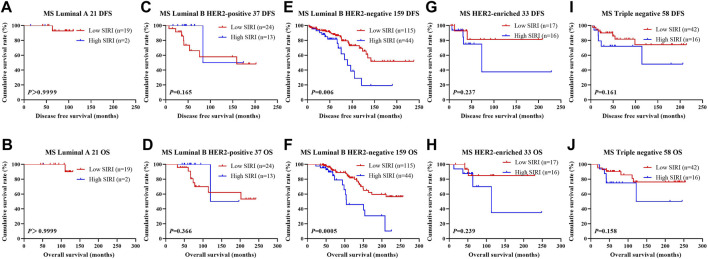
DFS and OS based on SIRI scores in patients with breast cancer of various molecular subtype (Non-NACT group). DFS and OS based on SIRI scores in patients with breast cancer of various molecular subtype (Non-NACT group). **(A)** Kaplan-Meier analysis of DFS for the SIRI of patients with luminal A breast cancer. **(B)** Kaplan-Meier analysis of OS for the SIRI of patients with luminal A breast cancer. **(C)** Kaplan-Meier analysis of DFS for the SIRI of patients with luminal B HER2-positive breast cancer. **(D)** Kaplan-Meier analysis of OS for the SIRI of patients with luminal B HER2-positive breast cancer. **(E)** Kaplan-Meier analysis of DFS for the SIRI of patients with luminal B HER2-negative breast cancer. **(F)** Kaplan-Meier analysis of OS for the SIRI of patients with luminal B HER2-negative breast cancer. **(G)** Kaplan-Meier analysis of DFS for the SIRI of patients with HER2-overexpressing breast cancer. **(H)** Kaplan-Meier analysis of OS for the SIRI of patients with HER2-overexpressing breast cancer. **(I)** Kaplan-Meier analysis of DFS for the SIRI of patients with triple-negative breast cancer. **(J)** Kaplan-Meier analysis of OS for the SIRI of patients with triple-negative breast cancer.

The relationship between SIRI and molecular type of breast cancer was assessed to ascertain the prognostic value of SIRI (shown in Figure 3, Figure 4, Figure 5). The log-rank analysis demonstrated that the average DFS and OS in the low SIRI group was drastically longer in contrast to patients with high SIRI scores.

### The Association Between Systemic Inflammatory Response Index Scores and Lymph Vessel Invasion

**FIGURE 6:**
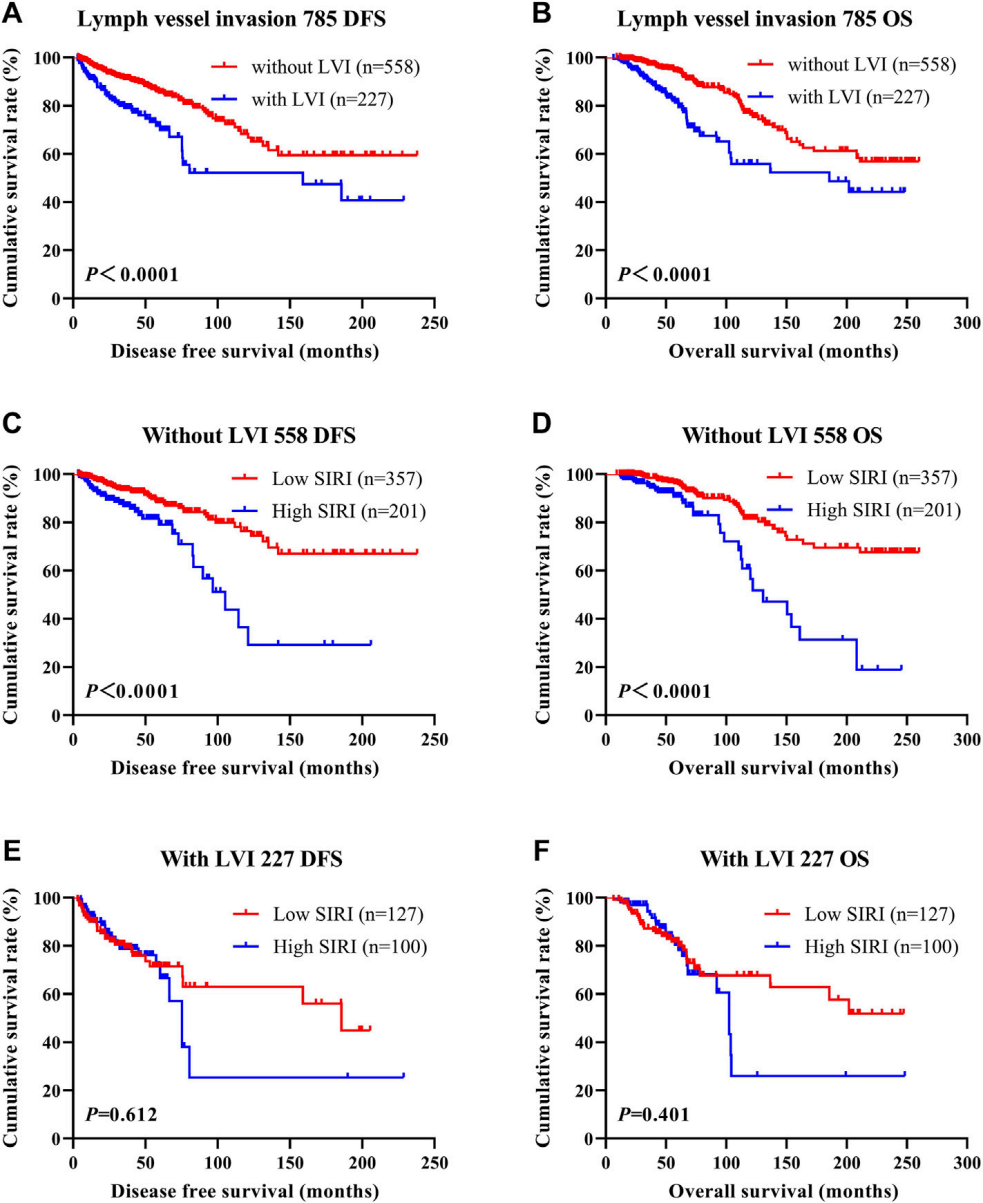
DFS and OS based on the presence of lymph vessel invasion in breast cancer patients. DFS and OS based on the presence of lymph vessel invasion in breast cancer patients. **(A)** Kaplan-Meier analysis of DFS for the SIRI of all patients with breast cancer. **(B)** Kaplan-Meier analysis of OS for the SIRI of all patients with breast cancer. **(C)** Kaplan-Meier analysis of DFS for the SIRI of breast cancer patients without lymph vessel invasion. **(D)** Kaplan-Meier analysis of OS for the SIRI of breast cancer patients without lymph vessel invasion. **(E)** Kaplan-Meier analysis of DFS for the SIRI of breast cancer patients with lymph vessel invasion. **(F)** Kaplan-Meier analysis of OS for the SIRI of breast cancer patients with lymph vessel invasion.

**FIGURE 7:**
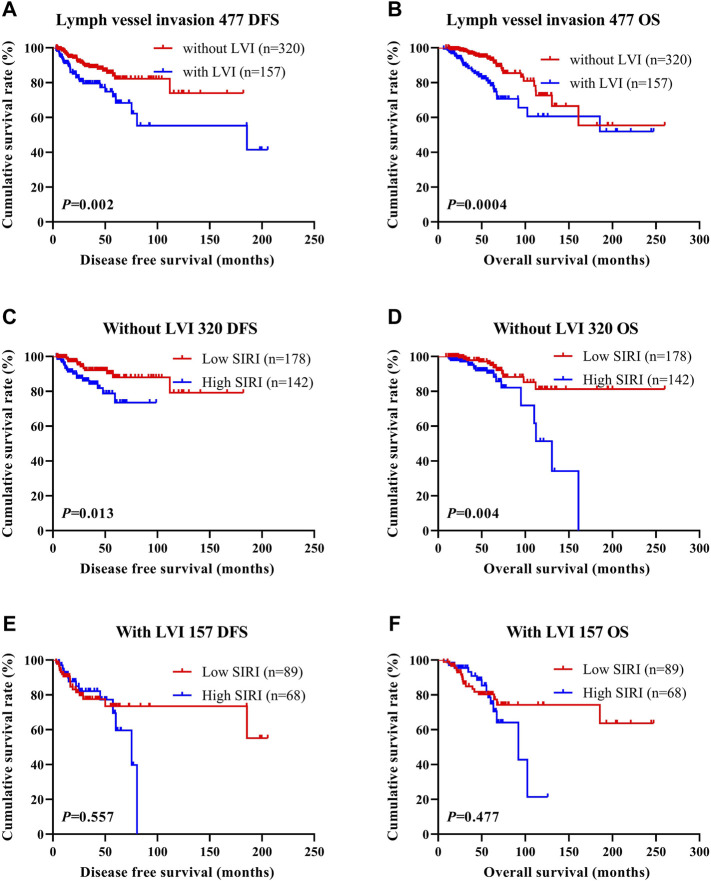
DFS and OS based on the presence of lymph vessel invasion in breast cancer patients (NACT group). DFS and OS based on the presence of lymph vessel invasion in breast cancer patients (NACT group). **(A)** Kaplan-Meier analysis of DFS for the SIRI of all patients with breast cancer. **(B)** Kaplan-Meier analysis of OS for the SIRI of all patients with breast cancer. **(C)** Kaplan-Meier analysis of DFS for the SIRI of breast cancer patients without lymph vessel invasion. **(D)** Kaplan-Meier analysis of OS for the SIRI of breast cancer patients without lymph vessel invasion. **(E)** Kaplan-Meier analysis of DFS for the SIRI of breast cancer patients with lymph vessel invasion. **(F)** Kaplan-Meier analysis of OS for the SIRI of breast cancer patients with lymph vessel invasion.

**FIGURE 8:**
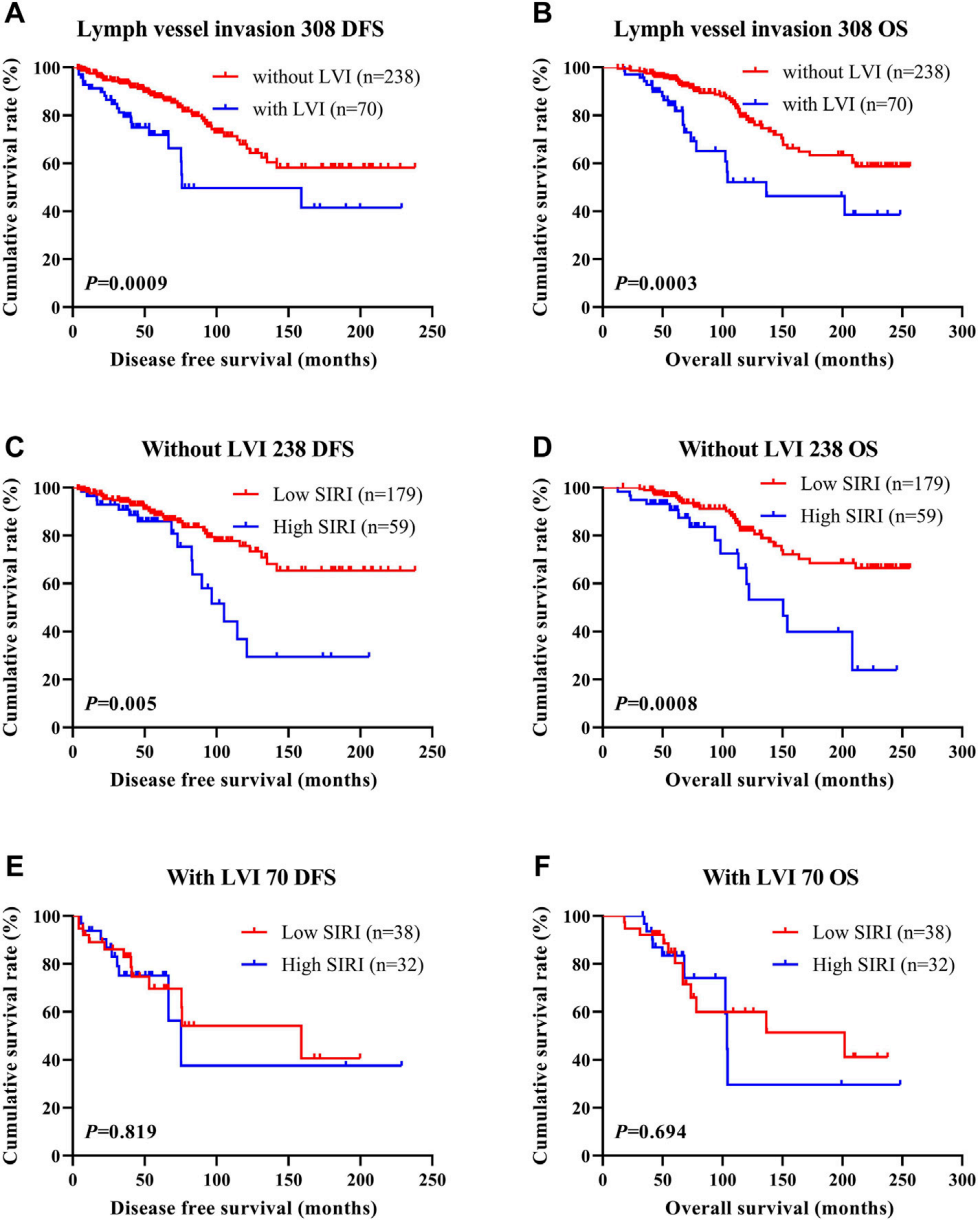
DFS and OS based on the presence of lymph vessel invasion in breast cancer patients (non-NACT group). DFS and OS based on the presence of lymph vessel invasion in breast cancer patients (non-NACT group). **(A)** Kaplan-Meier analysis of DFS for the SIRI of all patients with breast cancer. **(B)** Kaplan-Meier analysis of OS for the SIRI of all patients with breast cancer. **(C)** Kaplan-Meier analysis of DFS for the SIRI of breast cancer patients without lymph vessel invasion. **(D)** Kaplan-Meier analysis of OS for the SIRI of breast cancer patients without lymph vessel invasion. **(E)** Kaplan-Meier analysis of DFS for the SIRI of breast cancer patients with lymph vessel invasion. **(F)** Kaplan-Meier analysis of OS for the SIRI of breast cancer patients with lymph vessel invasion.

LVI was found to be an independent factor of DFS and OS based on univariate and multivariate analyses. Of the 785 cases of breast cancer, 227 cases were associated with LVI, and 558 cases were not. The relationship between SIRI and LVI was analyzed to determine the prognostic value of SIRI. The average DFS and OS in patients who did not have LVI were 50.96 and 79.65 months, respectively. The average DFS and OS in patients who had LVI were 28.97 and 53.37 months, respectively. Patients without LVI had notably longer mean DFS and OS in comparison to patients who had LVI (*χ* ^2^ = 20.940, *p* < 0.0001 and *χ* ^2^ = 26.540, *p* < 0.0001), as shown in Figure 6A and Figure 6B. Among the 558 patients without LVI, patients who had low SIRI scores had mean DFS and OS of 46.40 and 69.37 months, respectively; The average DFS and OS of high SIRI score patients were 30.00 and 54.43 months, respectively. Similarly, low SIRI group patients had notably longer mean DFS and OS in contrast to those with high SIRI scores, as evaluated using log-rank analysis (*χ* ^2^ = 16.020, *p* < 0.0001 and *χ* ^2^ = 22.050, *p* < 0.0001). Among the 227 patients with LVI, the mean DFS and OS were much longer in those with low SIRI scores in contrast to the high SIRI score group (*χ* ^2^ = 0.257, *p* = 0.612, and *χ* ^2^ = 0.705, *p* = 0.401), as shown in Figures 6C–F. The DFS and OS of SIRI and LVI of the NACT and non-NACT cohorts are shown in Figure 7 and Figure 8, respectively.

### The Association Between Systemic Inflammatory Response Index Scores and Neoadjuvant Chemotherapy/Postoperative Chemotherapy

**FIGURE 9:**
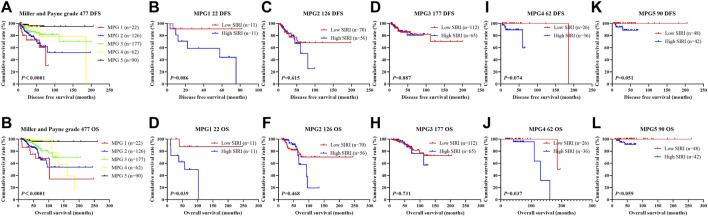
DFS and OS based on Miller and Payne grade (MPG) in breast cancer patients who received NACT. DFS and OS based on Miller and Payne grade (MPG) in breast cancer patients who received NACT. **(A)** Kaplan-Meier analysis of DFS based on MPG for the SIRI of patients with breast cancer. **(B)** Kaplan-Meier analysis of OS based on MPG for the SIRI of patients with breast cancer. **(C)** Kaplan-Meier analysis of DFS based on MPG1 for the SIRI of patients with breast cancer. **(D)** Kaplan-Meier analysis of OS based on MPG1 for the SIRI of patients with breast cancer. **(E)** Kaplan-Meier analysis of DFS for the SIRI of patients with breast cancer (MPG2). **(F)** Kaplan-Meier analysis of OS for the SIRI of patients with breast cancer (MPG2). **(G)** Kaplan-Meier analysis of DFS for the SIRI of patients with breast cancer (MPG3). **(H)** Kaplan-Meier analysis of OS for the SIRI of patients with breast cancer (MPG3). **(I)** Kaplan-Meier analysis of DFS for the SIRI of patients with breast cancer (MPG4). **(J)** Kaplan-Meier analysis of OS for the SIRI of patients with breast cancer (MPG4). **(K)** Kaplan-Meier analysis of DFS for the SIRI of patients with breast cancer (MPG5). **(L)** Kaplan-Meier analysis of OS for the SIRI of patients with breast cancer (MPG5).

In the NACT group, 141 patients underwent TP neoadjuvant chemotherapy, 28 patients received AC/ACF neoadjuvant chemotherapy, 223 patients received AT neoadjuvant chemotherapy, 27 patients received CT/ACT neoadjuvant chemotherapy, and 58 patients received other neoadjuvant chemotherapy regimens. All 477 patients received surgical treatment after neoadjuvant chemotherapy. 247 patients were not treated with postoperative chemotherapy, while 230 patients did. Of the 230 who received postoperative chemotherapy, 39 patients received TP chemotherapy, 37 patients received AT chemotherapy, 30 patients were treated with CT/ACT chemotherapy, 43 patients received AC/ACF chemotherapy, and 81 patients received other chemotherapy regimens. The clinical benefit rate (CR + PR + SD) was 98.53% (470/477), and the clinical objective response rate (CR + PR) was 66.88% (319/477). The MPG grade system was used to evaluate the pathological response of neoadjuvant chemotherapy. There were 22 MPG 1 cases (4.61%), 126 MPG 2 cases (26.42%), 177 MPG 3 cases (37.11%), 62 MPG 4 cases (13.00%), and 90 MPG 5 cases (18.87%). 72 cases (15.09%) achieved pCR, while 405 cases (84.90%) did not. The relationship between SIRI and MPG grade was analyzed to determine the prognostic value of SIRI. Log-rank analysis showed that mean DFS and OS were significantly different among various MPG grades (*χ* ^2^ = 18.290, *p* < 0.0001 and *χ* ^2^ = 18.020, *p* < 0.0001), as shown in Figure 9.

**FIGURE 10:**
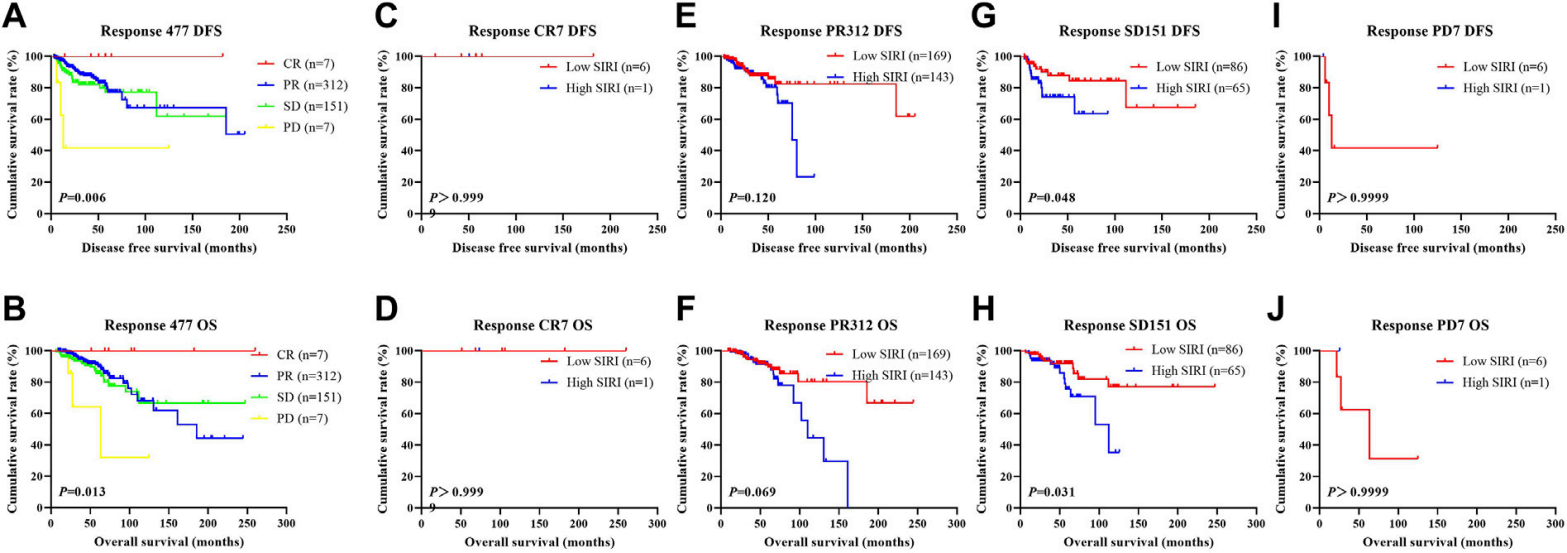
DFS and OS derived from response to neoadjuvant chemotherapy in breast cancer patient who received NACT. DFS and OS derived from response to neoadjuvant chemotherapy in breast cancer patient who received NACT. **(A)** Kaplan-Meier analysis of DFS for the SIRI of patients with breast cancer. **(B)** Kaplan-Meier analysis of OS for the SIRI of patients with breast cancer. **(C)** Kaplan-Meier analysis of DFS for the SIRI of patients with breast cancer. **(D)** Kaplan-Meier analysis of OS for the SIRI of patients with breast cancer. **(E)** Kaplan-Meier analysis of DFS for the SIRI of patients with breast cancer. **(F)** Kaplan-Meier analysis of OS for the SIRI of patients with breast cancer. **(G)** Kaplan-Meier analysis of DFS for the SIRI of patients with breast cancer. **(H)** Kaplan-Meier analysis of OS for the SIRI of patients with breast cancer. **(I)** Kaplan-Meier analysis of DFS for the SIRI of patients with breast cancer. **(J)** Kaplan-Meier analysis of OS for the SIRI of patients with breast cancer.

We further scrutinized how SIRI was related to response to neoadjuvant chemotherapy was scrutinized to determine the prognostic value of SIRI. Log-rank analysis demonstrated the average DFS and OS among different response groups were statistically significant (*χ* ^2^ = 12.540, *p* = 0.006 and *χ* ^2^ = 10.820, *p* = 0.013), as shown in Figure 10.

### The Association Between Systemic Inflammatory Response Index Scores and Chemotherapy Toxicity and Adverse Effects

Toxicity and adverse effects experienced by patients who received two cycles of NACT were evaluated. In the NACT group, common chemotherapeutic side effects included anorexia, alopecia, oral ulcers, diarrhea, vomiting, nausea, other gastrointestinal reactions, hepatic dysfunction, myelosuppression, thrombocytopenia, neutropenia, leucopenia, anemia, and peripheral neurotoxicity. There were no chemotherapy-related deaths during treatment. The degree of liver dysfunction was statistically different between the two groups (*χ* ^2^ = 7.146, *p* = 0.028) (Table 5).

**TABLE 5:** Correlation between SIRI and toxicity assessment.

Parameters	N	SIRI 477			
Cases (n)		Low SIRI 267	High SIRI 210	χ2	*p* value
Decreased appetite				1.825	0.177
No	70 (14.68%)	34 (12.73%)	36 (17.14%)		
Yes	407 (85.32%)	233 (87.27%)	174 (82.86%)		
Nausea				1.982	0.159
No	59 (12.37%)	28 (10.49%)	31 (14.76%)		
Yes	418 (87.63%)	239 (89.51%)	179 (85.24%)		
Vomiting				3.391	0.066
No	234 (49.06%)	121 (45.32%)	113 (53.81%)		
Yes	243 (50.94%)	146 (54.68%)	97 (46.19%)		
Diarrhea				0.286	0.593
No	444 (93.08%)	250 (93.63%)	194 (92.38%)		
Yes	33 (6.92%)	17 (6.37%)	16 (7.62%)		
Mouth ulcers				1.398	0.237
No	463 (97.06%)	257 (96.25%)	206 (98.10%)		
Yes	14 (2.94%)	10 (3.75%)	4 (1.90%)		
Alopecia				0.767	0.381
No	222 (46.54%)	129 (48.31%)	93 (44.29%)		
Yes	255 (53.46%)	138 (51.69%)	117 (55.71%)		
Peripheral neurotoxicity				2.559	0.110
No	390 (81.76%)	225 (84.27%)	165 (78.57%)		
Yes	87 (18.24%)	42 (15.73%)	45 (21.43%)		
Anemia				0.526	0.769
Grade 0	257 (53.88%)	144 (53.93%)	113 (53.81%)		
Grade 1–2	215 (45.07%)	121 (45.32%)	94 (44.76%)		
Grade 3–4	5 (1.05%)	2 (0.75%)	3 (1.43%)		
Leukopenia				1.138	0.566
Grade 0	138 (28.93%)	72 (26.97%)	66 (31.43%)		
Grade 1–2	233 (48.85%)	134 (50.19%)	99 (47.14%)		
Grade 3–4	106 (22.22%)	61 (22.85%)	45 (21.43%)		
Neutropenia				1.714	0.425
Grade 0	143 (29.98%)	76 (28.46%)	67 (31.90%)		
Grade 1–2	179 (37.53%)	107 (40.07%)	72(34.29%)		
Grade 3–4	155 (32.49%)	84 (31.46%)	71 (33.81%)		
Thrombocytopenia				0.553	0.758
Grade 0	372 (77.99%)	210 (78.65%)	162 (77.14%)		
Grade 1–2	98 (20.55%)	54 (20.22%)	44 (20.95%)		
Grade 3–4	7 (1.47%)	3 (1.12%)	4 (1.90%)		
Gastrointestinal reaction				1.485	0.476
Grade 0	38 (7.97%)	18 (6.74%)	20 (9.52%)		
Grade 1–2	433 (90.78%)	245 (91.76%)	188 (89.52%)		
Grade 3–4	6 (1.26%)	4 (1.50%)	2 (0.95%)		
Myelosuppression				0.357	0.836
Grade 0	90 (18.87%)	50 (18.73%)	40 (19.05%)		
Grade 1–2	175 (36.69%)	101 (37.83%)	74 (35.24%)		
Grade 3–4	212 (44.44%)	116 (43.45%)	96 (45.71%)		
Hepatic dysfunction				7.146	0.028
Grade 0	371 (77.78%)	196 (73.41%)	175 (83.33%)		
Grade 1–2	105 (22.01%)	70 (26.22%)	35 (16.67%)		
Grade 3–4	1 (0.21%)	1 (0.37%)	0 (0.00%)		

## Discussion

Breast cancer is a very common female malignancy whose incidence has surpassed that of lung cancer (Siegel et al., 2020). According to the 2020 World Health Organization (WHO) and International Agency for Research on Cancer (IARC) research, 19.29 million additional breast cancer cases are diagnosed every year. There are currently 2.26 million breast cancer cases worldwide, exceeding the 2.2 million cases of lung cancer (Siegel et al., 2020). Similar proportions are reported by the China National Cancer Center, which shows that China diagnoses 420,000 new female breast cancer patients every year, with 120,000 women dying from the disease. Patients are being diagnosed at an increasingly younger age, with mortality also increasing every year in spite of the current comprehensive breast cancer management protocols that involve surgery, supplemented by a combination of radiotherapy, chemotherapy, targeted therapy, and endocrine therapy (Tufano et al., 2021). At present, individualized treatment based on tumor characteristics, patient characteristics, and treatment response has emerged as the preferred means of treatment. These methods have greatly reduced patient mortality. Nevertheless, breast cancer is a heterogeneous disease with not all subtypes amenable to current therapies, cementing the position of this disease as the primary instigator of malignancy-associated mortalities in females around the world. NACT is an important part of systemic management of breast cancer, and is effective in reducing tumor size, clinical stage, improve surgical treatment outcomes while having an aesthetic effect (Colomer et al., 2019).

With the development of the field of tumor biology, several investigations have discovered that inflammation is involved in the initiation, development, and metastasis of tumors. Peripheral platelets, monocytes, lymphocytes, and neutrophils, are associated with the initiation and degree of inflammation (Xie et al., 2018). Many inflammatory markers have been used to predict the occurrence, progression, stage, and prognosis of tumors (Zhu et al., 2018). The reason may be that tumor tissues stimulate the proliferation of inflammatory cells in peripheral blood by secreting a number of pro-inflammatory substances (Li et al., 2018). Studies have confirmed cancer progression and recurrence are more likely to occur when the numbers of inflammatory cells such as neutrophils and monocytes in peripheral blood are relatively increased, and the numbers of immune cells such as lymphocytes and monocytes are relatively decreased (Qi et al., 2021). Inflammation directly brings about changes in the tumor microenvironment that directly promotes and augments malignant cellular transformation, invasion, and metastasis. A number of studies have shown that inflammatory markers in the tumor microenvironment can predict how breast cancer progresses along with its prognosis, with the inflammatory response representing an important marker of breast cancer outcomes. This carries significant implications regarding the role of inflammation in clinical disease assessment and treatment strategy formulation (Chen et al., 2020; Hua et al., 2020). Therefore, it is of great research significance to actively dissect the relationship between common peripheral blood markers and breast cancer patient prognosis.

Several cancers have demonstrated evidence of a systemic inflammatory response, although the exact cause of this phenomenon has not been completely reported (Topkan et al., 2020). Various inflammatory cells comprising of lymphocytes, monocytes, and neutrophils correlate to the prognosis of many tumors (Galdiero et al., 2018). Neutrophils augment tumor progression primarily by promoting the production of interleukin-6 (IL-6), arginase-1 (Arginase-1), and vascular endothelial growth factor (VEGF) (Corbeau et al., 2020). Lymphocytes are critical in tumor immune surveillance and are able to inhibit tumor progression and metastasis and directly kill tumor cells by stimulating natural killer cells (NK cells) and macrophages (Morrow et al., 2019). On the other hand, neutrophils inhibit lymphocytes, thereby inhibiting the anti-tumor immune response (Oba et al., 2021). Monocytes can differentiate into TAMs, and tumors secrete chemokines to recruit TAMs in the microenvironment. Some TAMs secrete growth factors and cytokines, promote angiogenesis, and facilitate immune escape, thus accelerating tumor progression (Olingy et al., 2019).

SIRI is an effective indicator of the immune status of malignant tumors that is established on peripheral venous lymphocyte, monocyte, and neutrophil counts (Wang et al., 2021). Research has revealed SIRI as an independent prognostic factor in several malignancies (Wei et al., 2020; Zhang et al., 2020). Hua et al. (2020) reported that SIRI was prognostic for postmenopausal breast cancer patients who undergo surgery, with patients with higher SIRI scores experiencing worse OS. Wang et al. (2020) used SIRI, histological grading, TNM stage, and a number of other indicators to build models that were able to predict 5-years and 10-years breast cancer survival rates. They found that the changes in SIRI scores 4 weeks after breast cancer surgery were correlated to survival. Breast cancer patients with more varied SIRI scores had worse overall survival (Wang et al., 2020). However, research on SIRI in breast cancer patients who undergo NACT treatment are scarce. Therefore, this study retrospectively studied the impact of SIRI on the survival and prognosis of breast cancer patients undergoing NACT.

This investigation outlines the relationship between SIRI and clinical pathology in breast cancer patients. A low SIRI score significantly influenced clinicopathological characteristics of patients, such as clinical data (BMI, US tumor size, US-LNM, clinical N, T, and overall TNM stages, postoperative chemotherapy regimen, operative time, postoperative chemotherapy and the frequency of treatment, postoperative targeted therapy), as well as nutritional and hematological parameters (LDH, CRP, CA125, FIB, INR, FDP, W, R, HB, N, L, M, B, and P). Univariate and multivariate analyses revealed that menopausal status, GLU, CA125, M, E, SIRI, histological grade, pathological N stage, Ki-67, CK5/6, LVI, postoperative chemotherapy, and postoperative targeted therapy were independent predictors of improved DFS and OS. The optimal threshold value for SIRI was 0.80, as determined using a ROC curve. The average DFS and OS survival times of those with low SIRI scores were notably prolonged (achieving statistical significance) compared to those with high SIRI scores.

We also scrutinized the association between SIRI scores and the pathological TNM stage. Data analyses revealed that the average DFS and OS in both early breast cancer and advanced breast cancer were longer in those in the low SIRI group in contrast to the high SIRI group, especially in advanced breast cancer. Similar findings were also seen in the NACT group, although the variability between the two cohorts was not significant. We also analyzed the relationship between SIRI and breast cancer molecular subtypes. There were differences in DFS and OS between high and low SIRI groups across all the analyzed molecular subtypes. While these differences were statistically significant in the three subtypes of Luminal B HER2-negative, HER2-overexpressed, and triple-negative breast cancer, no statistical significance was gained for the Luminal A type and Luminal B HER2-positive types.

Studies have pointed out that lymphatic vessel density and lymphatic infiltration are related to the prognosis of malignant tumors, with a higher degree of vascular infiltration conferring poorer patient prognosis (Wesch et al., 2014). Yamagata et al. (2021). reiterated that the presence of LVI was a crucial prognosticator in lymph node-positive breast cancer patients (Yamano et al., 2020). Our study also demonstrated that the DFS and OS of breast cancer patients with LVI were lower in contrast to those without LVI. Therefore, this study aimed to establish the association between SIRI and LVI. We found that the mean DFS and OS in breast cancer patients without LVI were longer in those with low SIRI scores compared to those with high SIRI scores. However, there was no significant variability between the two SIRI groups of breast cancer patients with LVI. For patients with LVI who received NACT, there was also no significant variability between in SIRI groups. We further assessed the relationship between SIRI, MPG, and response to chemotherapy. In different MPGs, the average DFS and OS survival times in patients with low SIRI scores were longer in contrast to those with high SIRI scores, although these differences failed to achieve statistical significance. In different responses, the average DFS and OS of the low SIRI group were longer compared to the high SIRI group (statistically significant). At the same time, we also analyzed the relationship between SIRI and the toxic side effects of NACT. Low SIRI scores correlated to improved liver function.

Many studies have described a robust inflammatory response to tumor occurrence and development. Quantifying the inflammatory response appears to be significant in clinical diagnosis as the degree of inflammation dictates the occurrence, progress, and outcomes of diseases. Neutrophils and monocytes both result from macrophage progenitor differentiation and possess similar roles in the inflammatory process. Both release a myriad of inflammatory mediators that includes the tumor necrosis factor, epidermal growth factor, and vascular endothelial growth factor; both promote tumor cell proliferation and blood vessel formation; both can inhibit the activity of T lymphocyte-mediated tumor escape from immune surveillance. Lymphocytes are also critical regulators of the tumor immune response and modulate the ability of tumors to hide from immune detection. The increase in the absolute value of neutrophils and monocytes and the decrease in the absolute value of lymphocytes in peripheral blood is associated with the occurrence, proliferation, and progression of tumors. SIRI takes into consideration peripheral blood neutrophils, lymphocytes, and monocytes to reflect the body’s inflammatory response. Therefore, SIRI can be used as a practical clinical indicator of tumor progression and prognosis. We previously noted that SIRI is not widely used as a prognostic indicator in breast cancer patients treated with neoadjuvant chemotherapy. China faces a problem of rising numbers of breast cancer patients. Coupled with the unequal distribution of healthcare resources in the country, the discovery of a commonly used, reproducible, and minimally invasive prognostic parameter that can also guide clinical management would greatly benefit breast cancer patients.

In conclusion, this investigation outlines the relationship between SIRI and breast cancer. Lower SIRI scores appear to confer a better prognosis in breast cancer. Nevertheless, our study is limited due to its small sample size and single-center origin. Future studies would benefit from multicenter patient data collection. The optimal threshold value of SIRI is related to the number of patients included and pathological conditions. Further studies are required to verify the SIRI threshold value of 0.80 that was obtained in this study.

## Data Availability

The original contributions presented in the study are included in the article/Supplementary Material, further inquiries can be directed to the corresponding authors.
